# STAT3 Undergoes Acetylation-dependent Mitochondrial Translocation to Regulate Pyruvate Metabolism

**DOI:** 10.1038/srep39517

**Published:** 2016-12-22

**Authors:** Yan S. Xu, Jinyuan J. Liang, Yumei Wang, Xiang-zhong J. Zhao, Li Xu, Ye-yang Xu, Quanli C. Zou, Junxun M. Zhang, Cheng-e Tu, Yan-ge Cui, Wei-hong Sun, Chao Huang, Jing-hua Yang, Y. Eugene Chin

**Affiliations:** 1 Cancer Research Center, Shandong University School of Medicine, Jinan, 250012, China; 2 The Key Laboratory of Stem Cell Biology, Institutes of Health Sciences, Shanghai Institutes for Biological Sciences, Chinese Academy of Sciences, 320 Yueyang Road, Shanghai 200031, China; 3 Department of Immune Diseases, School of Basic Medicine, Zhejiang Chinese Medical University, Hangzhou, Zhejiang 310053, China; 4 Translational Medicine Center, Shanghai Chest Hospital, Shanghai Jiaotong University School of Medicine, Shanghai 200032, China

## Abstract

Cytoplasmic STAT3, after activation by growth factors, translocates to different subcellular compartments, including nuclei and mitochondria, where it carries out different biological functions. However, the precise mechanism by which STAT3 undergoes mitochondrial translocation and subsequently regulates the tricarboxylic acid (TCA) cycle-electron transport chain (ETC) remains poorly understood. Here, we clarify this process by visualizing STAT3 acetylation in starved cells after serum reintroduction or insulin stimulation. CBP-acetylated STAT3 undergoes mitochondrial translocation in response to serum introduction or insulin stimulation. In mitochondria, STAT3 associates with the pyruvate dehydrogenase complex E1 (PDC-E1) and subsequently accelerates the conversion of pyruvate to acetyl-CoA, elevates the mitochondrial membrane potential, and promotes ATP synthesis. SIRT5 deacetylates STAT3, thereby inhibiting its function in mitochondrial pyruvate metabolism. In the A549 lung cancer cell line, constitutively acetylated STAT3 localizes to mitochondria, where it maintains the mitochondrial membrane potential and ATP synthesis in an active state.

Quiescent signal transducer and activator of transcription 3 (STAT3) in the cytoplasm is activated by cytokines or growth factors present in the cellular environment^1^. Cytokine- or growth factor-activated STAT3 proteins undergo post-translational modifications, including tyrosine and serine phosphorylation, acetylation, and methylation^2^,^3^,^4^,^5^. Tyr705/Ser727 phosphorylation in the C-terminal domain plays a critical role in STAT3 promoter binding and transcriptional activation within the nucleus^6^,^7^. Genes activated by phospho-STAT3 participate in diverse processes, including stem cell self-renewal growth, T cell differentiation, the cell cycle and metastasis^8^,^9^,^10^.

Ser727-phosphorylated STAT3 has been reported to undergo mitochondrial translocation^11^. In mitochondria, STAT3 is suspected to enhance electron respiratory chain activity and ATP production by interacting with complexes I and II and consequently increasing NADH^12^. Cells expressing mitochondrial localization signal (MLS)-STAT3 with an S727A substitution display decreased complex I activity in the electron transport chain (ETC) and increased reactive oxygen species (ROS) accumulation under hypoxic conditions, as compared with that observed in cells expressing MLS-STAT3^13^. STAT3 may be involved in Ras-dependent cellular transformation via augmented electron transport chain activity. However, direct protein interactions between STAT3 and complexes I/II are not required for optimal ATP production or oxidative phosphorylation *in vivo*^14^.

STAT3 shuttles between the cytoplasm and nucleolus in response to phosphorylation, and DNA binding and promoter initiation by nuclear STAT3 is terminated via dephosphorylation^6^,^15^,^16^,^17^. However, the mechanisms by which STAT3 shuttles between the cytoplasm and mitochondria and how mitochondrial STAT3 activity is reversed in mitochondria remain largely unknown. Interestingly, mitochondrial enzymes involved in removing posttranslational modifications catalyse deacetylation^18^, thus suggesting an important role of acetylation in mitochondrial activity. Three Sirt2 family members (SIRT3, SIRT4, and SIRT5) have been reported to reside in mitochondria^19^,^20^. Therefore, acetylation may be involved in energy metabolism, because acetylation often plays a critical role during cell starvation/release.

To examine whether STAT3 mitochondrial translocation occurs during starvation and release, as well as the role of STAT3 acetylation in mitochondrial function during metabolism, we analysed STAT3 activation by serum reintroduction or by insulin. Insulin regulates carbohydrate and fat metabolism by promoting the conversion of glucose to pyruvate^21^,^22^. We report here that both the N- and C-terminal domains of STAT3 are acetylated by CBP, a histone acetyltransferase (HAT), in serum-starved cells stimulated with insulin. We found that insulin-activated STAT3 undergoes acetylation-dependent mitochondrial translocation. Through the formation of a complex with pyruvate dehydrogenase complex E1 (PDC E1), an upstream component of the ETC, STAT3 elevates the mitochondrial membrane potential (MMP) and ATP production. SIRT5 deacetylates STAT3, thus disrupting its activity in mitochondria. In cancer cells with a low Warburg effect, STAT3 is constitutively acetylated and undergoes steady-state translocation into mitochondria, where it is constitutively involved in energy metabolism.

## Results

### Insulin-activated STAT3 is targeted to mitochondria via acetylation

**Figure 1:**
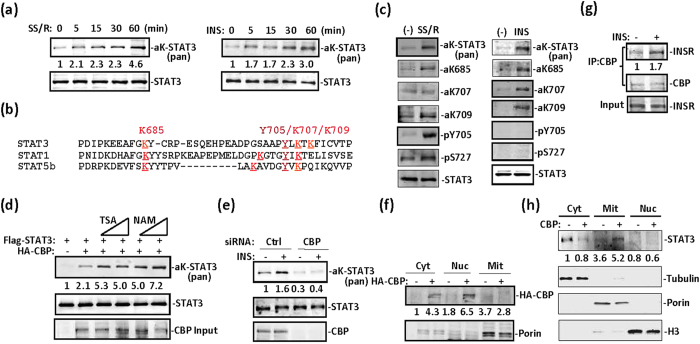
Growth factor induces STAT3 acetylation in serum-starved cells. (**a**) Time course of induction of STAT3 acetylation by serum starvation and reintroduction (SSR) (left) or serum starvation and insulin treatment (right) in PC3 cells transfected with STAT3. Pan acetyl-lysine antibody was used to detect acetyl-STAT3. (**b**) Alignment of C-terminal dimerization domain sequences from STAT1, STAT3, and STAT5b. K685, K707, and K709 are acetylation sites identified by mass spectrometry analysis of STAT3 prepared from PC3 cells transfected with STAT3. Y705 is phosphorylated after cytokine treatment. (**c**) SSR for 30 min (left) or insulin (5 μg/ml) for 15 min (right) induced STAT3 post-translational modifications, as indicated in MEFs that were serum-starved (−) for at least 6 hrs. (**d**) 293T cells were transfected with STAT3 or STAT3 and CBP, and this was followed by a 6-hr treatment with NAM (100 μM, 200 μM) or TSA (1 μM, 5 μM). Whole cell lysates were prepared and subjected to western blotting with the indicated antibodies. Acetyl-STAT3 was detected with anti-pan acetyl-lysine antibody. (**e**) CBP was depleted with siRNA in 293T cells. After insulin treatment for 30 min, WCL were prepared, and acetyl-STAT3 was blotted with the anti-pan acetyl-lysine antibody. (**f**) In 293T cells, CBP was transfected for 36 hrs before harvesting. Cytoplasmic, mitochondrial, and nuclear fractions were prepared for CBP and mitochondrial marker porin analysis with specific antibodies using western blotting. (**g**) MEFs were treated with insulin for 30 min or were left untreated. CBP was immunoprecipitated and subjected to blotting with anti-insulin receptor or anti-CBP antibodies. (**h**) In 293T cells, CBP was transfected for 36 hrs before harvest. Cytoplasmic, mitochondrial and nuclear fractions were prepared for STAT3 analysis.

We and others have previously reported that STAT3 is acetylated at multiple sites after IL-6-type cytokine stimulation^3^,^23^,^24^. Here, we subjected serum-starved fibroblast cells to serum starvation and reintroduction or to insulin treatment for varying time periods. A similar pattern of STAT3 acetylation induction was observed under both conditions (Fig. 1a). A number of conserved lysine residues flanking the most important phospho-tyrosine residue of the C-terminal domain have been previously reported by different groups^25^. From mass spectrometry analysis of STAT1, STAT3 and STAT5b mutants in PC3 cells, we confirmed three crucial acetylation sites, K685, K707 and K709, flanking the phospho-tyrosine 705 in the C-terminal domain (Fig. 1b). We prepared a number of antibodies against acetyl-STAT3, including those against acetyl-K87, acetyl-K707, and acetyl-K709 residues (Supplementary Fig. 1a). Although serum starvation- and reintroduction-activated STAT3 was moderately phosphorylated on both Y705 and S727 sites (Fig. 1c, left), insulin treatment showed no effect on STAT3 Y705 or S727 phosphorylation (Fig. 1c, right). In MEFs, serum starvation and reintroduction or insulin treatment induced STAT3 acetylation on K685, K707, and K709, as confirmed by using these antibodies against acetyl-STAT3 (Fig. 1c).

CBP overexpression induced STAT3 acetylation, and both nicotinamide (NAM) and tricostatin A (TSA) treatments induced STAT3 acetylation (Fig. 1d), thus suggesting that both HDAC and Sirtuin family members catalyse STAT3 deacetylation in cells. CBP deletion abolished insulin-induced STAT3 acetylation in MEFs, thus suggesting that CBP is critical for STAT3 acetylation (Fig. 1e). We observed CBP in both cytoplasmic and nuclear fractions but not in the mitochondrial fraction in 293T cells (Fig. 1f). CBP was associated with the insulin receptor, and insulin treatment enhanced the association in MEFs (Fig. 1g), as did exogenously expressed CBP and insulin receptor in 293T cells (Supplementary Fig. 2a). Interestingly, CBP overexpression induced STAT3 mitochondrial translocation (Fig. 1h).

**Figure 2:**
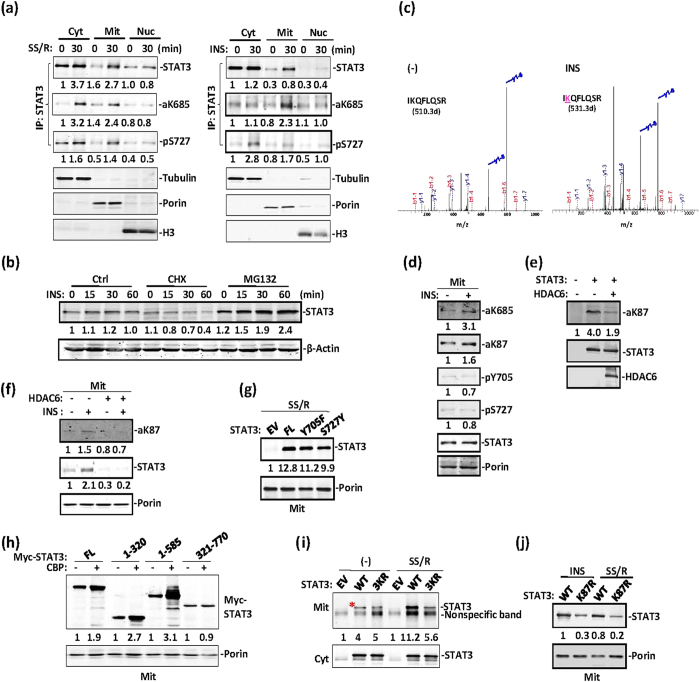
STAT3 undergoes acetylation-dependent mitochondrial translocation. (**a**) All MEFs were serum starved for 6 hrs before treatment with insulin (right) or SSR (left) for 30 min. Cell lysates of the cytoplasm (Cyt), mitochondria (Mit) and nuclei (Nuc) were prepared for western blotting analysis of STAT3 and other subcellular fraction markers. (**b**) MEFs were pretreated with cycloheximide or MG132, then subjected to insulin treatment for the indicated times. STAT3 or β-actin levels were analysed in the whole cell lysates by using western blotting. (**c**) Mass spectrometric analysis of STAT3 immunoprecipitated from STAT3-expressing 293T cells treated with insulin or left untreated. K87 was identified as the lysine residue of STAT3 that was acetylated after insulin treatment. (**d**) The mitochondrial fraction was prepared from MEFs treated with insulin or left untreated. Mitochondrial STAT3 was then analysed by western blotting. (**e**) Myc-tagged STAT3 was transfected alone or along with HDCA6 in 293T cells. Immunoprecipitated STAT3 was blotted with anti-aK87-STAT3 or anti-STAT3 antibodies, and HDAC6 was detected in the input. (**f**) Immunoprecipitated STAT3 was blotted with anti-aK87-STAT3 or anti-STAT3 in mitochondrial lysates from the above types of 293T cells. (**g**) 293T cells were transfected with an EV, wild-type Myc-STAT3, Myc-STAT3-Y705F and S727A mutants. Mitochondrial lysate was prepared under conditions of SSR for 30 min. STAT3 was analysed by western blotting with an anti-Myc antibody. (**h**) Myc-tagged full-length STAT3 or different domains, as indicated, were transfected alone or along with CBP in 293T cells. Mitochondrial lysates were prepared for STAT3 and porin blotting analysis. (**i**) PC3 cells were transfected with an EV, wild-type Myc-STAT3 or Myc-STAT3-3KR mutant, and treated with SSR for 30 min. STAT3 in cytoplasmic and mitochondrial lysates fractions was analysed by western blotting with an anti-Myc antibody. (**j**) 293T cells were transfected with wild-type Myc-STAT3 and Myc-STAT3-K87R mutant. Mitochondrial lysate was prepared under conditions of SSR or insulin treatment for 30 min.

We then compared STAT3 subcellular localization in response to serum starvation and reintroduction and insulin treatment. Serum starvation and reintroduction activated STAT3 proteins translocated to the mitochondria, and a small fraction translocated into the nucleus (Fig. 2a, left). STAT3 underwent mitochondrial translocation primarily in response to insulin treatment (Fig. 2a, right). Both serum starvation and reintroduction and insulin activated-STAT3 were K685 acetylated, whereas STAT3 S727 phosphorylation was observed primarily under the condition of serum starvation and reintroduction (Fig. 2a). To exclude the possibility that STAT3’s mitochondrial accumulation was due to enhanced STAT3 protein synthesis or stability in response to insulin treatment, we treated MEFs with insulin in the presence of the protein biosynthesis inhibitor cycloheximide (CHX) or the proteasome inhibitor MG132. As expected, in the presence of CHX, STAT3 protein levels were moderately and gradually decreased, whereas in the presence of MG132, elevated STAT3 protein levels were not affected after insulin treatment in cells (Fig. 2b).

We next purified STAT3 from insulin-treated STAT3-expressing 293Ts and performed mass spectrometry analysis. Insulin-activated STAT3 was acetylated on K87 as well as at the aforementioned C-terminal sites (K685, K707, and K709) (Fig. 2c, Supplementary Fig. 1b). No tyrosine/serine-phosphorylated peptides were recovered. STAT3 was acetylated not only in the C-terminal domain, as we and others have previously reported, but also in the N-terminal domain^3^,^26^. Utilizing a specific polyclonal antibody against acetyl-K87 of STAT3 (Supplemental Fig. 1a), we clearly detected STAT3 K87 acetylation in mitochondrial lysates prepared from insulin-treated MEFs (Fig. 2d) or from CBP-transfected 293T cells (Supplementary Fig. 2b). The HDAC6 deacetylation of STAT3 on K87 outside of the mitochondria (Fig. 2e) was in agreement with previous reports that STAT3 is deacetylated by HDACs in the cytoplasm^27^, and HDAC6 weakened STAT3 mitochondrial translocation induced by insulin (Fig. 2f).

To delineate the mechanism by which STAT3 translocates to mitochondria after insulin stimulation, we constructed both C-terminally and N-terminally truncated STAT3. However, neither N-terminal nor C-terminal deletion affected STAT3’s mitochondrial translocation (Supplementary Fig. 2c). Hence, these terminal regions do not contain the mitochondrial localization signal (MLS) for STAT3. Moreover, phosphorylation on Y705 and S727 as a result of serum starvation and reintroduction also had no obvious effects on STAT3 mitochondria location (Fig. 2g). To define the domain of STAT3 interacts with CBP, we co-transfected various STAT3 domains along with CBP. The STAT3 N-terminal domain (residues 1–320) but not the STAT3 C-terminal domain (residues 321–770) underwent enhanced mitochondrial translocation in response to CBP, whereas the N-terminal 321–770 truncation translocated to mitochondria independently of CBP (Fig. 2h). Thus, acetylation dramatically enhanced STAT3 mitochondrial translocation, and an acetylation motif within the N-terminal domain is responsible for this activity. STAT3 with K685R, K707R or K709R mutations (3KR) still underwent mitochondrial translocation in response to serum reintroduction or insulin treatment (Fig. 2i). In contrast, the STAT3 K87R mutant, compared with wild-type STAT3, exhibited decreased mitochondrial translocation (Supplementary Fig. 2d), even after insulin treatment or serum starvation and reintroduction (Fig. 2j). These results strongly suggest that STAT3 undergoes both acetylation-dependent and acetylation-independent mitochondrial translocation.

### STAT3 forms a complex with PDC E1 and is crucial for pyruvate-to-acetyl-CoA conversion

**Figure 3:**
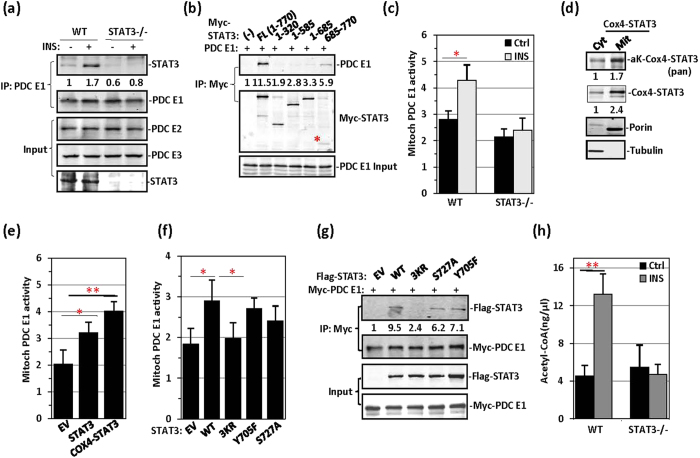
STAT3 promotes the conversion of pyruvate to acetyl-CoA by PDC. (**a**) Wild-type and STAT3−/− MEFs were treated with insulin for 30 min, and this was followed by immunoprecipitation of PDC E1. PDC E1 precipitates were subjected to western blotting for STAT3 co-immunoprecipitation. PDC E2 and PDC E3 were detected in the cell lysates. (**b**) Myc-STAT3 full length (FL) and Myc-STAT3 domains, as indicated, were cotransfected with PDC E1 in 293T cells, which were then subjected to by anti-Myc immunoprecipitation. STAT3 FL and STAT3 685-770 precipitated STAT3. (**c**) PDC E1 activity was measured in WT and STAT3−/− MEFs treated with insulin or left untreated. (**d**) 293T cells were transfected with an EV or Myc-tagged STAT3 fused with Cox4 MLS (Cox4-STAT3). Cytoplasmic (cyt) and mitochondrial lysates (mit) were analysed for Cox4-STAT3 acetylation and Cox4-STAT3 (anti-Myc) via western blotting. (**e**) Mitochondrial PDC E1 activity was measured in STAT3−/− MEFs transfected with an EV, Myc-STAT3, or Myc-Cox4-STAT3. (**f**) Mitochondrial PDC E1 activity was measured in STAT3−/− MEFs transfected with STAT3 WT, STAT3-3KR, STAT3-Y705F, or STAT3-S727A mutant. (**g**) Flag-STAT3 WT and Flag-STAT3 mutants, as indicated, were transiently transfected along with Myc-tagged PDC E1 in 293T cells. Anti-Myc immunoprecipitates were analysed for Flag-STAT3. (**h**) Acetyl-CoA content was measured in WT or STAT3−/− MEFs treated with insulin.

To confirm that STAT3 is a component of the respiratory complex, we performed mass spectrometry analyses of the binding partners in STAT3 immunoprecipitates prepared from HeLa cell mitochondria. Although complex I/II components and GRIM-19 have been reported to be STAT3 binding proteins in mitochondria^11^,^12^, our mass spectrometric analysis did not identify these proteins as STAT3 binding factors. A number of mitochondrial proteins, including PDC E1, cytochrome C, and SIRT5, were all identified as STAT3 binding proteins. In MEFs, interaction between STAT3 and PDC E1 was induced after insulin treatment (Fig. 3a). In STAT3−/− MEFs, the expression levels of PDC components were not altered (Fig. 3a), thus suggesting that STAT3 knockout did not affect the expression of PDC components, including PDC E1, dihydrolipoyl transacetylase (PDC E2) and dihydrolipoyl dehydrogenase (PDC E3). In 293T cells, the STAT3 C-terminal domain was involved in the PDC E1 interaction (Fig. 3b).

Given the critical role of PDC E1 in the conversion of pyruvate to acetyl-CoA, we analysed the effect of STAT3 on PDC E1 activity in MEFs. STAT3−/− MEFs failed to induce PDC E1 activity in response to insulin (Fig. 3c). To estimate mitochondrial STAT3 activity, we constructed Cox4-STAT3 by fusing the MLS (yeast oxidase complex IV nucleotide sequence) to the N-terminus of STAT3; both the mitochondrial localization and acetylation Cox4-STAT3 were higher than that observed in the cytoplasm (Fig. 3d). When Cox4-STAT3 was overexpressed in STAT3−/− MEFs, Cox4-STAT3 was more active than wild-type STAT3 in PDC E1 activity induction (Fig. 3e), presumably because of more efficient mitochondrial translocation and acetylation by Cox4-STAT3 (Supplementary Fig. 2e). Among the STAT3 mutants overexpressed in STAT3−/− MEFs, the 3KR mutant significantly inhibited PDH activity compared with wild-type STAT3 and Y705F and S727A mutants (Fig. 3f). This inhibition can be explained by the poor interaction between the 3KR STAT3 mutant and PDC E1 (Fig. 3g). The STAT3 S727A mutation also affected the interaction with PDC E1 (Fig. 3f,g). Given that the PDC E1 complex is responsible for pyruvate-to-acetyl-CoA conversion, we assayed mitochondrial acetyl-CoA levels. As expected, acetyl-CoA levels were not responsive to insulin treatment in STAT3−/− MEFs (Fig. 3h). Thus, STAT3 most probably affects mitochondrial activity at the pyruvate-to-acetyl-CoA conversion step.

### STAT3 is deacetylated by multiple SIRTs in mitochondria

**Figure 4:**
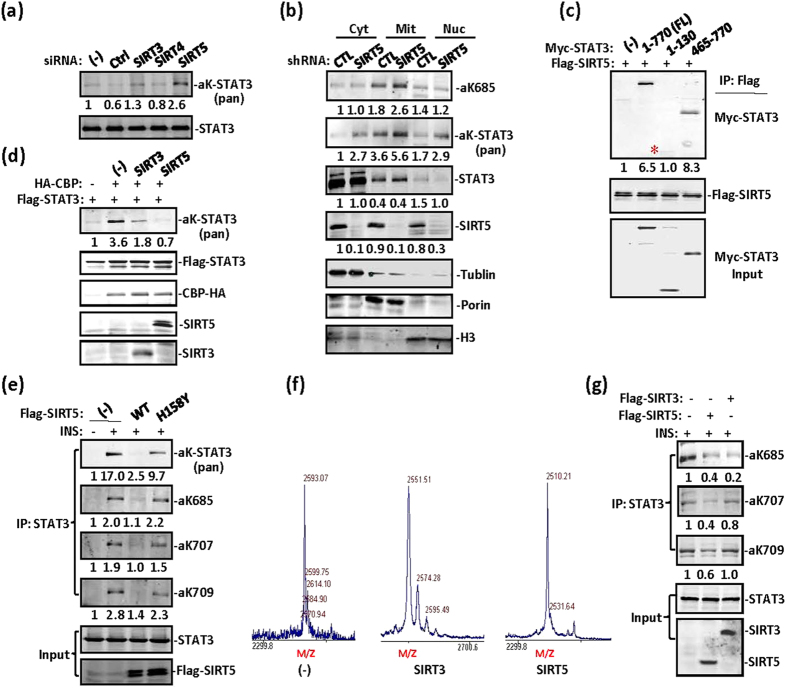
SIRT5 is responsible for STAT3 deacetylation in mitochondria. (**a**) In HeLa cells SIRT3, SIRT4, and SIRT5 were depleted with siRNA. Immunoprecipitated STAT3 was detected with anti-pan-acetyl-lysine antibody via western blotting. (**b**) A549 cells were transfected with control or SIRT5 shRNA. Elevated STAT3 acetylation was detected in cytoplasmic, mitochondrial, and nuclear fractions prepared from these cells. Tubulin, porin, and histone H3 were used as cytoplasmic, mitochondrial, and nuclear controls, respectively. (**c**) In 293T cells, STAT3 full length, 1–130, and 465–770 were cotransfected with Flag-SIRT5. STAT3 association was detected in Flag-SIRT5 immunoprecipitates via western blotting. (**d**) In 293T cells, Flag-STAT3 was cotransfected with an EV, CBP, CBP and SIRT3 or CBP and SIRT5. Acetylation of immunoprecipitated STAT3 was then detected with a pan-acetyl-lysine antibody. (**e**) In MEFs, STAT3 was cotransfected with Flag-SIRT5 WT or Flag-SIRT5 H158Y mutant, then subjected to insulin treatment for 30 min. STAT3 acetylation or phosphorylation was detected with the indicated antibodies. (**f**) A synthetic acetyl-peptide covering the STAT3 K685 site was incubated with Flag-SIRT5 or Flag-SIRT3 protein purified from 293T cells and NAD. Mass spectrometry analysis was then performed. (**g**) MEFs were transiently transfected with SIRT3 or SIRT5. STAT3 C-terminal acetylation induced by insulin was analysed with specific antibodies against STAT3 acetylation by western blotting, as indicated. SIRT3 and SIRT5 were blotted with anti-Flag; STAT3 was detected in the input.

Given the positive effect of NAM on STAT3 acetylation in Fig. 1d, we screened Sirtuin family members for STAT3 deacetylation activity in HeLa cells by performing siRNA depletion. SIRT5 depletion and to a lesser extent SIRT3 depletion enhanced STAT3 acetylation in cells (Fig. 4a), thus suggesting that both SIRT3 and SIRT5 are responsible for STAT3 deacetylation in HeLa cell mitochondria. In A549 cells, acetyl-STAT3 was constitutively detected in lysates, and SIRT5 depletion enhanced STAT3 acetylation in the cytoplasm, mitochondria, and nucleus (Fig. 4b). To identify the domains of STAT3 involved in the interaction with SIRT5, we constructed STAT3 domains and tested their interaction with SIRT5. Whereas the C-terminal domain 465–770 of STAT3 strongly interacted with SIRT5, the N-terminal 1–130 also showed a weak interaction with STAT3 (Fig. 4c). CBP-overexpression-induced STAT3 acetylation was abolished by transient transfection with SIRT5 (Fig. 4d). Insulin-induced STAT3 acetylation in MEFs was completely inhibited by overexpression of wild-type SIRT5 but not the mutant SIRT3-H158Y (Fig. 4e). SIRT5 is responsible for the deacetylation of various mitochondrial proteins^28^,^29^,^30^,^31^. Our *in vitro* assay results also revealed that both SIRT3 and SIRT5 deacetylated STAT3 on K685 (Fig. 4f), whereas SIRT3, unlike SIRT5, was unable to deacetylate STAT3 on lysine 707 and 709 (Fig. 4g). STAT3 was deacetylated in mitochondria by both SIRT5 and to a much lesser extent by SIRT3. Hence, in the subsequent studies, we studied SIRT5’s deacetylation of STAT3 in mitochondria. We concluded that STAT3 acetylation is a reversible process and that both SIRT5 and SIRT3 are responsible for STAT3 deacetylation in mitochondria even though these two SIRT family members have opposite roles in mitochondrial metabolism^32^,^33^,^34^,^35^. The significance of STAT3 deacetylation by these two SIRT family members remains largely unknown. The role of SIRT3 in STAT3 deacetylation is still under investigation.

### Acetyl-STAT3 affects TCA-respiratory chain function

**Figure 5:**
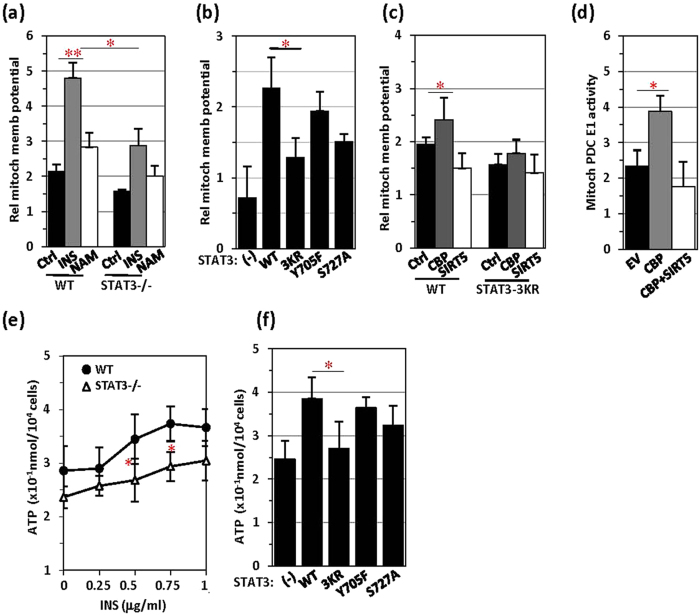
Mitochondrial membrane potential and ATP generation are inhibited by SIRT5. (**a**) Wild-type or STAT3−/− MEFs were treated with insulin (5 μg/ml, 30 min) or NAM (100 μM, 6 hrs) and subjected to mitochondrial membrane potential analysis with JC-1 staining. (**b**) The mitochondrial membrane potential was measured in STAT3−/− MEFs transfected with STAT3 WT or mutants as indicated. (**c**) STAT3 WT or 3KR mutant MEFs were transfected with an EV, CBP and CBP together with SIRT5, and the mitochondrial membrane potential was measured. (**d**) PDC E1 activity (unit/10^4^ cells) was measured in STAT3−/− MEFs transfected with an EV, CBP and CBP together with SIRT5. (**e**) ATP production was compared between STAT3 WT and STAT3−/− MEFs in response to treatment with insulin at the indicated concentrations. (**f**) In STAT3−/− MEFs, different forms of STAT3 were transiently transfected, and ATP levels were analysed.

Because mitochondria are the powerhouses that are crucial in TCA-ETC regulation, we analysed the effect of STAT3 on mitochondrial TCA-ETC function. To effectively monitor the mitochondrial membrane potential, we stained mitochondria with the mitochondrial membrane potential dye JC-1 and quantitated the staining signals. The mitochondrial membrane potential was dramatically enhanced by insulin treatment or, to a much lesser extent, by NAM treatment (Fig. 5a). In STAT3−/− MEFs, both the basal level and the insulin response of the mitochondrial membrane potential were greatly decreased (Fig. 5a). PC3 cells are a prostate cancer cell line bearing a STAT3 whole-gene-deletion mutation on chromosome 17^36^. In these STAT3-null cells, we compared the effects of STAT3 mutations on mitochondrial membrane potential. STAT3 with the 3KR mutation markedly affected the mitochondrial membrane potential, and STAT3 with the S727A mutation affected mitochondrial membrane potential activity to a lesser extent (Fig. 5b), thus suggesting that serine-phosphorylation plays a less important role than acetylation in STAT3-mediated changes in mitochondrial membrane potential^13^. The STAT3-mediated elevation of the mitochondrial membrane potential responded to CBP transfection but was inhibited by SIRT5 transfection (Fig. 5c). As expected, CBP transfection increased the STAT3-mediated induction of PDC activity, whereas SIRT5 cotransfection abolished this effect (Fig. 5d).

The positive effect of acetyl-STAT3 on mitochondrial TCA-ETC function was further confirmed by examining ATP generation in cells. Insulin-induced ATP production was compared in wild-type and STAT3−/− MEFs. STAT3−/− MEFs generated less ATP than did control MEFs (Fig. 5e). STAT3−/− MEFs did not respond to insulin (up to 1 μg/ml) in terms of ATP generation, even though the basal level of ATP production was relatively high in these cells (Fig. 5e). We then expressed STAT3 mutants in STAT3−/− MEFs and compared their effects on ATP generation in response to insulin treatment. Whereas the Y705F mutation showed no apparent effect on STAT3-mediated ATP generation, the 3KR mutation and to a lesser extent the S727A mutation abolished STAT3-enhanced ATP production (Fig. 5f). Therefore, acetyl-STAT3 affects ATP generation and the membrane potential in mitochondria, presumably by affecting PDC activity in acetyl-CoA conversion.

**Figure 6:**
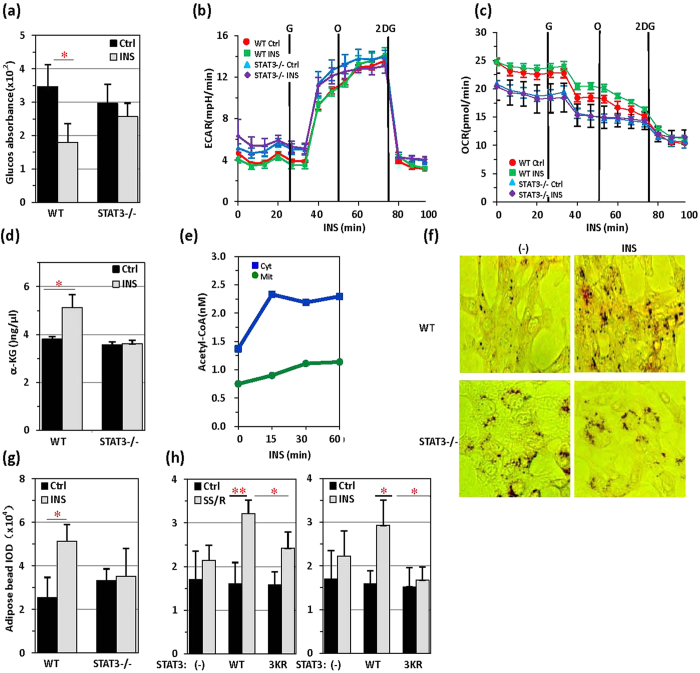
Insulin triggers conversion of glucose to fatty acids. (**a**) Cellular glucose levels were measured in control or STAT3−/− MEFs receiving insulin (5 μg/ml) treatment for 30 min or no treatment. (**b**) Seahorse extracellular acidification rates (ECAR) were measured in quadruplicate wells containing equal numbers of wild-type or STAT3−/− MEFs treated with insulin (5 μg/ml). (**c**) The Seahorse oxygen consumption rate (OCR) was measured in quadruplicate wells containing equal numbers of wild-type or STAT3−/− MEFs treated with insulin (5 μg/ml). (**d**) α-ketoglutaric acid concentrations were measured in control or STAT−/− MEFs receiving insulin (5 μg/ml) treatment for 30 min or no treatment. (**e**) Cytoplasmic and mitochondrial Acetyl-CoA levels were measured in MEFs treated with insulin for various times as indicated. (**f**) Oil red O staining of control MEFs and STAT3−/− MEFs, treated with or without insulin (5 μg/ml, 30 min). Lipid droplet accumulation was visualized with a microscope. (**g**) The IOD (integrated optical density) of the lipid droplets from (**c**) was measured with IPP software (**d**). (**h**) The IOD (integrated optical density) of lipid droplets was measured in STAT3−/− MEFs stained with Oil red O after SSR (90% DMEM and 10% FBS, 30 min), insulin (5 μg/ml, 30 min) and NAM (100 μM, 6 hrs) treatment.

The major effect of insulin on cellular sugar metabolism is acceleration of sugar clearance in cells. STAT3 was indispensable for insulin regulation of cellular sugar metabolism, because cellular glucose levels were rapidly decreased by insulin treatment in wild-type but not STAT3−/− MEFs (Fig. 6a). However, STAT3 depletion did not affect glycolysis, as revealed by extracellular acidification rates (ECAR) detected by using a Seahorse XF96 extracellular flux analyser, a result consistent with our conclusion that STAT3 regulates glucose metabolism in mitochondria (Fig. 6b). The oxygen consumption rate (OCR) was clearly higher in wide-type MEFs treated with insulin than in STAT3−/− MEFs in the same experimental process (Fig. 6c). Comparatively, insulin-induced TCA upregulation was affected, because insulin did not induce an increase in α-ketoglutaric acid (α-KG) levels in STAT3−/− MEFs (Fig. 6c). Insulin-induced acetyl-CoA production was significantly increased in both the cytoplasmic and mitochondrial fractions (Fig. 6d). Mitochondrial acetyl-CoA primarily participates in downstream metabolic pathways such as cellular respiration and the TCA cycle. Acetyl-CoA transferred to the cytoplasm is involved in lipid synthesis. Intracellular lipids were quantified by Oil red O staining. In wild-type MEFs, insulin induced rapid Oil red O accumulation or lipid envelope formation, whereas in STAT3−/− MEFs, constitutive Oil red O accumulation or lipid envelope formation was not detected (Fig. 6e,f). Both serum starvation and reintroduction and insulin treatment dramatically increased adipose accumulation when wild-type STAT3 but not STAT3 with the 3KR mutation was introduced into STAT3−/− MEFs (Fig. 6g). Thus, whereas STAT3 is absolutely required for mitochondrial activity in promoting TCA function, STAT3 also sensitizes the conversion of glucose to fatty acids in response to insulin treatment in cells.

**Figure 7:**
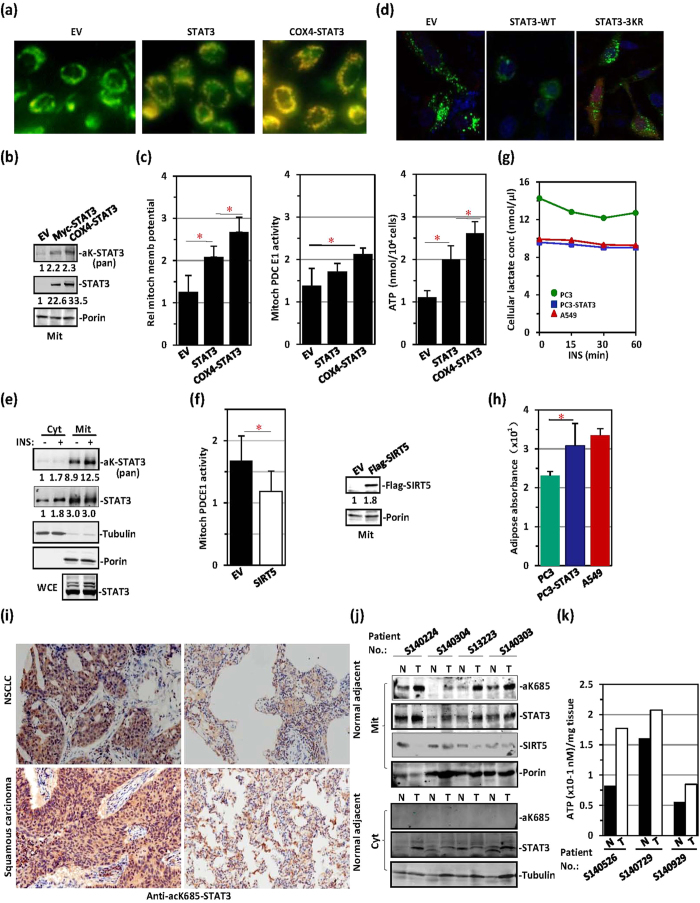
STAT3 constitutively translocates to mitochondria and affects TCA-respiratory chain function in cancer cells. (**a**) PC3 cells (STAT3-null) were transfected with an EV, STAT3 or Cox4-STAT3 and visualized with the mitochondrial membrane potential dye JC-1. (**b**) Mitochondrial lysates from the above three types of PC3 cells were prepared for STAT3 acetylation analysis. (**c**) Mitochondrial membrane potential (left), mitochondrial PDC E1 activity (middle) and ATP production levels (right) were measured in the above three types of PC3 cells. (**d**) Parental PC3 cells (STAT3-null), PC3 cells expressing STAT3 WT, or PC3 cells expressing STAT3-3KR mutant were transfected with GFP-LC3 (green), stained with DAPI (blue), and subjected to confocal microscopy. (**e**) A549 cells were treated with or without insulin. STAT3 acetylation was detected with anti-pan-acetyl-lysine antibody in cytoplasmic and mitochondrial fractions. (**f**) Mitochondrial PDC E1 activity was measured in A549 cells transfected with an EV or Flag-SIRT5. Flag-SIRT5 levels (right) were detected via western blotting in these cells. (**g**) Lactate levels were measured in parental PC3 (green), PC3-STAT3 (blue) or A549 cells (red) after insulin treatment for the indicated times. (**h**) The absorbance of adipose droplets stained by Oil red O was measured in parental PC3 (green), PC3-STAT3 (blue) or A549 cells (red) at 490 nm in extracts prepared with isopropanol. (**i**) Lung tissue samples (cancer sections and adjacent normal sections) obtained from non-small cell adenocarcinoma and squamous carcinoma lung cancer patients were immunostained with polyclonal antibody against K685-acetylated STAT3. (**j**) Mitochondrial and cytoplasmic fractions were prepared from tumour tissue samples (T) and adjacent normal tissue samples (N) from 4 lung cancer patients. aK685-STAT3, STAT3, SIRT5 and porin were analysed in the mitochondrial fraction (upper panel), aK685-STAT3, STAT3 and tubulin were analysed in the cytoplasmic fraction (lower panel). (**k**) ATP production was compared between tumour tissue samples (T) and adjacent normal tissue samples (N) from 3 lung cancer patients.

We next examined the status of mitochondrial STAT3 in cancer cells. PC3 cells exhibited a very low mitochondrial membrane potential, presumably because of a lack of STAT3 expression (Fig. 7a). We tagged STAT3 with Cox4 MLS-peptide, which increased STAT3 mitochondrial translocation, as previously reported (Fig. 7b)^37^. The mitochondrial membrane potential was enhanced after transfection with STAT3 and was markedly enhanced after transfection with Cox4-MLS-tagged STAT3 (Fig. 7a). In PC3 cells transfected with STAT3 or Cox4-MLS-tagged STAT3, PDC activity and ATP production were both elevated (Fig. 7c). STAT3 has previously been reported to block autophagy in cancer cells^38^,^39^,^40^,^41^. In PC3 cells, wild-type STAT3 but not 3KR-mutated STAT3 blocked autophagy, as reflected by the disappearance of LC3-GFP puncta (Fig. 7d). However, STAT3 was constitutively acetylated in the A549 lung adenocarcinoma cell line (Fig. 7e), and constitutive localization of acetyl-STAT3 to mitochondria was detected (Fig. 7e). Elevated PDC E1 activity was observed in these cells (Fig. 7f). Some cancer cells exhibit enhanced conversion of glucose into lactate via pyruvate, in the so-called Warburg effect. We therefore assayed lactate levels in these cancer cells. Lactate levels were much higher in PC3 cells than in A549 cells (Fig. 7g). However, the introduction of STAT3 into PC3 cells greatly decreased the Warburg effect, as reflected by reduced lactate levels (Fig. 7g) and elevated glucose-to-fatty-acid metabolism (Fig. 7h). Immunohistochemical analyses showed that K685-acetylated STAT3 was widely distributed in cytoplasmic regions in cancer sections obtained from both non-small cell adenocarcinoma and squamous carcinoma lung cancer patients (Fig. 7i). In contrast, only sporadic staining of K685-acetylated STAT3 was observed in the adjacent normal lung tissue (Fig. 7i). To confirm that cytoplasmic K685-acetylated STAT3 was localized to mitochondria, we separated the mitochondrial fraction from the cytoplasm in cancer and adjacent normal tissue samples obtained from lung cancer patients. Subsequent western blotting revealed that levels of K685-acetylated STAT3 were higher in the mitochondria of cancer tissue sections than in normal tissue sections (Fig. 7j). In contrast, the SIRT5 level was lower in cancer tissue sections than in adjacent normal tissue sections (Fig. 7j). ATP production was also higher in cancer tissue sections than normal tissue sections, as expected (Fig. 7k). Therefore, the activation of mitochondrial metabolism by constitutively acetylated STAT3 may be critical in cancer development^42^,^43^,^44^.

## Discussion

Whereas the shuttling of STAT3 between the cytoplasm and nucleus depends exclusively on tyrosine and serine phosphorylation^45^,^46^, STAT3 mitochondrial translocation can be both acetylation-dependent and acetylation-independent, as demonstrated here. The S727A mutation has previously been shown to decrease mitochondrial STAT3 accumulation^11^. Our results revealed that insulin-activated STAT3 was mainly acetylated and undergoes mitochondrial rather than nuclear translocation. Moreover, the N-terminal region of STAT3 was responsible for CBP acetylation-dependent mitochondrial translocation. CBP-acetylated proteins are often involved in protein shuttling between the cytoplasm and subcellular organelles, protein-plasma membrane association and dissociation, and protein-protein interaction^47^,^48^,^49^. One plausible explanation is that acetylation neutralizes the positive charges of protein lysine residues, thus enabling the protein to move more flexibly across the negatively charged lipid bilayer membrane. Although GRIM19 and MLS-lacking STAT3 protein have been reported to exhibit mitochondrial colocalization^11^,^12^,^13^,^14^,^15^,^16^,^17^,^18^,^19^,^20^,^21^,^22^,^23^,^24^,^25^,^26^,^27^,^28^,^29^,^30^,^31^,^32^,^33^,^34^,^35^,^36^,^37^,^38^,^39^,^40^,^41^,^42^,^43^,^44^,^45^,^46^,^47^,^48^,^49^,^50^,^51^, STAT3 and GRIM19 complex formation has been shown to be independent of S727 phosphorylation, and neither STAT3 nor GRIM19 bears a typical MLS^50^.

Larner’s group has reported that STAT3 modulates complex I and II in the electron transport chain through an unknown mechanism^12^,^13^. GRIM-19 is a subunit of the mitochondrial NADPH:ubiquinone oxidoreductase complex. However, GRIM19 largely inhibits STAT3 transcription by disrupting STAT3-DNA binding activity and further inhibits STAT3-mediated cell proliferation^50^. Pyruvate dehydrogenase complex-E2 (PDC-E2) has previously been reported to interact with STAT family members in a cytokine stimulation-dependent manner^52^. CBP-acetylated STAT3 translocates into mitochondria where STAT3 associates with PDC E1 and promotes pyruvate oxidation. The N-terminal region of STAT3 is the coiled-coil domain, which is homologous among many structural proteins, enzymatic cofactors, as well as transcription factors^53^,^54^,^55^. The exceptionally tight packing of the coiled-coil domain is favourable for the precise control of enzymatic reactions. Proteins with STAT3-like linker-SH2 domains have been identified in plants and C. elegans^56^. These proteins are not conserved in their DNA binding domains, thus indicating that they may have additional roles beyond serving as transcription factors. Hence, by forming a complex with PDC, STAT3 may facilitate pyruvate-to-acetyl-CoA conversion by functioning as an enzymatic cofactor.

Unlike dephosphorylation, deacetylation can be readily executed within mitochondria, and many deacetylases are found in mitochondria. In mitochondria, SIRT3, SIRT4, and SIRT5 are responsible for the deacetylation of different substrates and the termination of their activities is involved in different steps of the TCA-ETC pathway^34^,^57^,^58^. STAT3 is an immediate substrate of SIRT5, thus suggesting that STAT3 may indeed be involved in multiple steps of the TCA-ETC pathway. In cancer or other metabolic disorders in which mitochondrial SIRT activity is downregulated, mitochondrial proteins may display constitutive or hyperacetylation. Constitutively acetylated STAT3 translocates into mitochondria, where it sustains altered TCA-ETC function for glycolytic and oxidative phosphorylation reactions. The STAT3-dependent metabolic regulation of pyruvate conversion in mitochondria supports Ras-dependent malignant transformation^59^.

Overall, acetylation-dependent STAT3 activity can be more readily manipulated than phosphorylation in mitochondria, owing to the presence of powerful deacetylation enzymes. This study highlights a role of acetyl-STAT3 in regulating pyruvate metabolism for TCA-ETC function in mitochondria. The involvement of STAT3 in energy metabolism via acetylation suggests that STAT3 may play a role in various tumour mechanisms.

## Materials and Methods

### Cell culture and reagents

PC3, A549, HeLa and 239T cells were obtained from the American Type Culture Collection and grown according to American Type Culture Collection recommendations. All of the above cell lines, as well as STAT3−/− and control MEFs (obtained from X.Y. Fu), were cultured in 90% DMEM, 10% FBS (Gibco, #10099-141), 100 μg*/*ml penicillin (Life Technologies, #15140-155) and 100 μg*/*ml streptomycin (Life Technologies, #15140–122) at 37 °C and 5% CO_2_.

Chemicals, including NAM (A2984), TSA (T1952), DAPI (5D8417), Oil red O (O0625), insulin (I3536), a glucose assay kit (GAGO20), an α-ketoglutaric acid assay kit (MAK054), acetyl-CoA assay kit (MAK039), and a lactate assay kit (MAK064), were obtained from Sigma. Mito-Tracker Green (C1048), JC-1 a mitochondrial membrane potential detection kit (C2006), a cytosolic protein extraction kit (P0027), a mitochondria extraction kit (C3601) and an ATP assay kit (S2006) were purchased from Beyotime (Shanghai). The pyruvate dehydrogenase activity assay kit was purchased from YoYongBio (Shanghai). The ELISA acetyl-CoA assay kit was purchased from Xinfan Biotechnology Co., Ltd. (Shanghai).

The anti-VDAC1/Porin (ab 14734) antibody was purchased from Abcam. The anti-H3 antibody (3638), anti-pS727-STAT3 antibody (9136) and anti-acK685-STAT3 antibody (2523) were from Cell Signaling Technology (Boston). The anti-STAT3 antibody (SC-482), anti-pY705-STAT3 antibody (SC-7993) and IgG (SC-3888, SC-3877) were from Santa Cruz Biotechnology (Santa Cruz). The anti-Flag antibody (F9291), anti-tubulin antibody (T2200) and IgG (15381) were from Sigma. Polyclonal antibodies to acetyl-STAT3 (acK87, acK707, acK709) were prepared by AB-land Biotech (Hangzhou). IgG controls for all immunoprecipitation and co-immunoprecipitation experiments are provided in Supplementary Fig. 1c.

### Human tissue acquisition

Human lung cancer tissue samples and adjacent normal tissue samples were procured from the Translational Medicine Center, Shanghai Chest Hospital. Experimental protocols were approved by both Shanghai Jiaotong University and Shandong University Institutional Review Boards (IRB). Methods described in this section were performed in accordance with all human research guidelines and regulations. Informed consent was obtained from all subjects in this study.

### Preparation of nuclear, cytosolic and mitochondrial protein extracts

Conventional whole-cell lysates, as well as cytoplasmic and nuclear fractions, were prepared as previously described^60^. Mitochondrial extracts were prepared using a mitochondrial extraction kit (Beyotime, #C3601), per the manufacturer’s instructions. Briefly, 5 × 10^6^ cells were routinely ground with a glass homogenizer (KIMBLE CHASE, #885302) in an ice bath for 22 strokes. Cytoplasmic, mitochondrial and nuclear fractions were separated through differential centrifugation (600 g, 10 min, 4 °C and 10,000 g, 10 min, 4 °C). The supernatant (cytosolic fraction) and pellet (mitochondrial fraction) were collected, and the pellet was further lysed to yield the final mitochondrial lysate. To confirm that pure extracts were obtained, the mitochondrial, nuclear and cytoplasmic fractions were separated by SDS-PAGE, and the presence of mitochondrial porin, nuclear H3, or cytoplasmic tubulin was demonstrated by western blot analysis using monoclonal antibodies.

Four groups of tumour and normal samples were prepared for tissue mitochondria extraction by using a tissue mitochondria extraction kit (Beyotime, #C3606). Briefly, 300 mg of tumour and normal tissue were washed 3 times with PBS, sheared as much as possible, and ground with 15 strokes of a glass homogenizer in an ice bath. Cytoplasmic and mitochondrial fractions were separated through differential centrifugation (600 g, 5 min, 4 °C and 10,000 g, 10 min, 4 °C). The supernatant (cytosolic fraction) and pellet (mitochondrial fraction) were collected, and the pellet was further lysed to yield the final mitochondrial lysate. The extracted proteins were prepared for subsequent western blotting analysis.

### Western blotting analysis

Cells exposed to different conditions were lysed in western and IP lysate buffer (Beyotime, #P0013J) containing 20 mM Tris (pH 7.5), 150 mM NaCl, and 1% Triton X-100 and supplemented with protease and phosphatase inhibitor cocktail (Thermo Scientific, #78440). Proteins were separated by SDS-PAGE and transferred to a nitrocellulose membrane (Millipore, # HATF00010). Membranes were blocked with 5% BSA in Tris-buffered saline and incubated with primary antibodies overnight. Blots were developed with a peroxidase-conjugated fluorescent secondary antibody for 1 h, and then the western blots were scanned and analysed on a LI-COR system (Odyssey). Full scans of the western blots in all figures are provided in Supplementary Fig. 3.

### SiRNA transfection

Small interfering RNAs (siRNAs) against PR, STAT3, SIRT3, SIRT4, and SIRT5 mRNAs were synthesized by Dharmacon, Inc. (Lafayette, CO). A non-silencing siRNA oligonucleotide from Dharmacon, which does not target any known mammalian genes, was used as a negative control. Transfection of siRNA duplexes was performed by using Lipofectamine 2000 (Invitrogen, #11668-027) transfection reagent, per the manufacturer’s directions.

### Mass spectrometry analysis (in-solution trypsin digestion)

Approximately 2 × 10^7^ 293T cells that had been transfected with STAT3 or other plasmids for 48 hrs were lysed in RIPA lysate solution (50 mM Tris-HCl, pH 7.4, 1%NP-40, 0.5% sodium deoxycholate, 0.1%SDS, 150 mM NaCl). Protein samples were adjusted to a known concentration in 100 mM NH_4_HCO_3_ buffer, and the pH was adjusted to 8.0 with 1 M Tri-HCl buffer (pH 8.0). Trypsin solution (w/w, Sigma, #T6567) was added to the protein samples (1:25) and incubated overnight at 37 °C. The trypsin lysates were added to an Amicon Ultra-0.5 ml Centrifugal Filter Unit (Ultracel-10K) and centrifuged at 13,000 rpm at 4 °C for 10 min, and the pellets were dried with a Speed Vac. Samples were resuspended in buffer A (deionized water with 0.1% formic acid); the peptide concentration was measured, and samples were used for LC-MS/MS analysis.

### cDNA reconstruction

We reconstructed STAT3 N-terminally fused with yeast cytochrome C oxidase 4 mitochondrial localization sequence (MLS), whose nucleotide sequence is ATGTTGTCTAGATATCTGCTTCGTCATTGTTCTCGTTCCCTTTCTTCCT TGTTTCTTTTTCTGCACAATATTTCAAGCTATACCAAGCATACAATCAA GGAATTCACAATGTTGTCTAGATATCTGCTTCAGCAAAAACCCGTGGT GAAAACTGCCCAAAACTTAGCAGAAGTTAATGGTCCAGAAACTTTGAT TGGTCCTGGTGCTAAAGAGGGTACCGTTCCAACAGACCTAGATCAAG AAACTGGTTTAGCTAGGTTAGAATTATTGGGTAAATTAGAGGGTATCGAT.

### Immunofluorescence staining and confocal microscopy

PC3 cells were cotransfected by GFP-LC3 with an empty vector, wild-type STAT3 or STAT3 3KR mutant for 24 hrs, and then subjected to serum starvation with RPMI-1640 for 12 hrs. DAPI staining was performed on PC3 cells on glass coverslips for 5 min, and cells were fixed with 4% paraformaldehyde for 30 min at room temperature. After being washed with PBS three times, coverslips were mounted in Citifluor (Amersham Biosciences), and stained cells were analysed on a Zeiss LSM 700 confocal microscope.

#### Tissue array immunostaining

The paraffin-embedded tissue microarray blocks were sectioned at 4-mm thickness, deparaffinized in xylene, and rehydrated in graded ethanol solution, and endogenous peroxidase activity was blocked by incubation with 3% H_2_O_2_ for 30 min at room temperature. Then, sections were immersed in citrate-NaOH buffer (10 mM sodium citrate, pH 7.0) for 40 min at 92 °C for restoration of antigenicity. The rehydrated sections were incubated overnight at 4 °C with anti-Lys685 (1:20, Abcam). The sections incubated with the first antibody were washed with Tris-buffered saline and were then incubated with a MaxVision HRP-Polymer anti-Rabbit IHC Kit (Maxin, Fuzhou) for 20 min at room temperature. The sections were visualized using a DAB Detection Kit (Maxin, Fuzhou) reaction followed by counterstaining with haematoxylin. Negative control experiments were performed by omitting the primary antibody.

### Mitochondrial PDC E1 activity analysis

Mitochondrial PDC E1 activity was assayed using a PDC E1 assay kit according to the manufacturer’s protocol (YoYongBio). Briefly, 5 × 10^6^ cells treated as indicated were prepared for mitochondrial extraction. Cells were fixed with 1 ml of reagent-1 and 10 μl of reagent-3, ground with a glass homogenizer (KIMBLE CHASE, #885302) 16 times in an ice-bath, and centrifuged at 600 g, 4 °C for 5 min. The supernatant was transferred to another 1.5-ml centrifuge tube and centrifuged at 10000 g, 4 °C for 10 min. A total of 200 μl of reagent-2 and 2 μl of reagent-3 were added to the pellet (mitochondria); ultrasonic fragmentation was performed in an ice bath (20% power, in 30 cycles of 3 sec on and 10 sec off); and PDC E1 activity was then detected at 605 nm on a microplate reader. Briefly, 50 μl of sample was added into 90 μl of working solution in a cuvette and percussion mixed. The initial absorbance value A1 at 605 nm was recorded immediately, and the absorbance value A2 was recorded after 1 min, with ΔA = A1 − A2. PDC E1 catalyses the dehydrogenation of pyruvate and simultaneously deoxidizes 2,6-DCPIP, thus resulting in a decrease in absorbance at 605 nm. U represents one PDH activity unit, which is defined as the consumption of 1 nmole of 2,6-DCPIP within 1 min by 1 × 10^4^ cells.

Calculation formula: PDH activity (U/10^4^ cell) = [ΔA × Vtr ÷ (ε × d) × 10^9^] ÷ (500 × Vs ÷ Vts) ÷ T = 0.366 × ΔA (Vtr: total reaction volume, ε: 2,6-two dichloroindophenol molar extinction coefficient, d: cuvette light path, Vs: sample volume, Vts: total sample volume).

### Mitochondrial membrane potential detection

MEFs and PC3 cells seeded in 12-well cell culture plates were treated as described and then stained with JC-1 according to the manufacturer’s instructions. Briefly, 6 × 10^5^ cells were harvested, fixed with 0.5 ml of cell culture media and 0.5 ml of JC-1 staining working solution, and incubated in a cell culture cabinet for 20 min after gentle shaking. Cells were collected through centrifugation at 600 g, 4 °C for 3 min and then washed 3 times with 1x JC-1 buffer solution. The cell fluorescence intensity was measured with a microplate reader in 96-well plates after cells were resuspended in 100 μl of 1x JC-1 buffer solution. The red (λex = 529 nm, λem = 590 nm) and green (λex = 470 nm, λem = 529 nm) average fluorescence intensities were measured on the microplate reader.

### Adipose drop staining

MEFs, A549 cells, and PC3 cells seeded in 12-well cell culture plates were treated with drugs and stained with Oil red O according to the manufacturer’s instructions (Sigma, #O0625). Briefly, after removal of the culture media, the cells were rinsed twice with PBS, after which 1 ml of fixative solution (10% formaldehyde) was added to cover cells for at least 1 hr at room temperature. After two gentle washes with 60% isopropanol, freshly prepared Oil red O working solution (60% Oil red O mixed with 40% deionized water) was added and incubated for 20 min at room temperature after the isopropanol had dried completely. The staining solution was removed, and the cells were immediately washed four times with deionized water. At this stage, microscopy images were collected to visualize pink to red oil droplets in cells. A total of 100 μl of dye-extraction solution (100% isopropanol) was added to each well for 5 min, and the absorbance was read at 490 nm in a 96-well plate on a microplate reader.

### ATP levels

MEFs or PC3 cells seeded in 12-well dishes were transfected with an empty vector (EV), STAT3 WT, Cox4-STAT3 or STAT3 mutants for 48 hrs or treated with different drugs. Cellular ATP content was measured according to the instruction manual for the reagent (Beyotime, #S2006). Cells were lysed with 100 μl lysis buffer from the assay kit. The supernatant was collected after centrifugation at 12000 g, 4 °C for 5 min and used to detect ATP levels; 50 μl of ATP detection working solution was added to each tube before detection for 5 min to completely consume the original ATP. The firefly luciferase activity of each sample, expressed in RLU (relative light unit), was measured for at least 2 sec with a GLOMA luminometer from Promega, with 20 μl of cell lysate added to each tube. A standard curve was constructed using 0.01, 0.03, 0.1, 0.3, 1.0, 3.0 and 10 μM ATP.

### Glucose consumption

Glucose consumption in cells was tested according to the manufacturer’s protocols (Sigma, #GAGO-20). Glucose was extracted from approximately 1 × 0^7^ MEFs with 2 ml deionized water. The solution was heated (<75 °C) to aid extraction. At time zero, the reaction was started by addition of 2.0 ml of assay reagent to the first tube (containing 1 ml of sample solution) and mixing. A 30-to-60-sec interval was allowed between additions of assay reagent to each subsequent tube. Reactions in each tube reacted for exactly 30 min at 37 °C and were stopped at 30–60 sec intervals by addition of 2.0 ml of 12 N H_2_SO_4_ (Sigma, #258105), and each tube was then mixed thoroughly. The absorbance of each tube was measured against a reagent blank at 540 nm on a microplate reader. The volumes of glucose standard solution used to generate the standard curve was 0, 0.02, 0.04, 0.06, and 0.08 ml.

### α-KG content

α-KG content was tested according to the manufacturer’s protocols (Sigma, #MAK054). Briefly, 2 × 10^6^ MEFs were homogenized in 100 ml ice-cold α-KG buffer. Samples were centrifuged at 13,000 g for 10 min to remove insoluble material, adjusted to a final volume of 50 ml with α-KG assay buffer, and deproteinized with a 10 kDa MWCO (Millipore) spin filter before addition to the reaction, to prevent interference from enzymes in the samples. Reactions consisted of 20 μl sample solution, 24 μl α-KG assay buffer, 2 μl α-KG converting enzyme, 2 μl α-KG development enzyme mix and 2 μl fluorescent peroxidase substrate, and were incubated at 37 °C for 30 min. The absorbance of each reaction system was measured at 540 nm (A_570_) on a microplate reader. A total of 10 μl of 100 mM α-KG standard solution was diluted with 990 μl water to prepare a 1 mM standard solution. The volumes 1 mM α-KG standard solution used to generate the standard curve were 0, 2, 4, 6, 8, and 10 μl.

### Acetyl-CoA content

For the fluorescence assay method, acetyl-CoA content was tested according to the manufacturer’s protocols (Sigma, #MAK039). Briefly, 1 × 10^7^ MEFs were frozen rapidly (liquid N2) and pulverized. Samples were deproteinized by PCA precipitation; 2 ml 1 N perchloric acid (PCA) (Sigma, #34288) was added to the sample while the sample was kept cold. The sample was then homogenized thoroughly and centrifuged at 13,000 g for 10 min to remove insoluble material. The supernatant was neutralized with 3 M potassium bicarbonate solution (Sigma, # 60339), added in aliquots of 1 ml/10 ml of supernatant during vortexing, until bubble evolution ceased (2–5 aliquots). The samples were then cooled on ice for 5 min, and the pH was verified to be in the range of 6–8 in 1 ml of sample. Samples were spun for 2 min to pellet potassium bicarbonate. Reactions consisted of 20 μl sample solution, 21 μl acetyl-CoA assay buffers, 2 μl acetyl-CoA substrate mix, 1 μl conversion enzyme, 5 μl acetyl-CoA enzyme mix, and 2 μl fluorescent probe and were incubated at RT for 10 min. Fluorescence intensity was measured (λex = 535/λem = 587 nm) in black, 96-well flat-bottom plates with clear bottoms. A total of 10 μl of 10 mM α-KG standard solution was diluted with 990 μl of water to prepare a 0.1 mM standard solution, and 100 μl of 0.1 mM standard solution was diluted with 400 μl of water to prepare a 0.02 mM standard solution. The volumes of the 0.02 mM acetyl-CoA standard solution used to generate the standard curve were 0, 10, 20, 30, 40, and 50 μl.

For the ELISA method, 1 × 10^7^ MEFs treated with insulin for 0, 15, 30, 60 min were separated into cytoplasmic and mitochondrial fractions. A total of 40 μl of sample dilution and 10 μl of sample were added to ELISA plates prepared according to the kit instructions, mixed gently, and incubated at 37 °C after plates were sealed with a sealing membrane. The liquid was discarded, and each well was washed 5 times for 30 sec each with 1x washing liquid. Next, 50 μl of enzyme labelling reagent was added to each well, and this was followed by incubation, as was described above. Then, 50 μl of chromogenic agent A and 50 μl chromogenic agent B were added and mixed gently, and the ELISA plates were placed at 37 °C for 15 min in the dark. A total of 50 μl of stop reagent was added to each reaction to terminate the reactions. Absorbance was measured at 450 nm in 96-well flat-bottom plates. The concentrations of the standard solution used to generate the standard curve were 25 pmol/L, 50 pmol/L, 100 pmol/L, 200 pmol/L, and 400 pmol/L.

### Lactate concentration

Lactate concentrations were detected according to the manufacturer’s protocols (MAK064). Briefly, 2 × 10^6^ PC3 cells or A549 cells were homogenized in 4 volumes of lactate assay buffer and centrifuged at 13,000 g for 10 min to remove insoluble material. A master reaction mix containing 20 μl sample solution, 26 μl lactate assay buffer, 2 μl lactate enzyme mix and 2 μl lactate probe was added, and reactions were incubated at RT for 30 min. Sample absorbance was measured at 570 nm (A_570_) on a microplate reader. A total of 10 μl of 100 nmole/μl lactate standard was diluted with 990 μl lactate assay buffer to generate a 1 nmole/μl standard solution. The volumes of the 1 nmole/μl lactate standard solution used to generate the standard curve were 0, 2, 4, 6, 8, and 10 μl.

### Seahorse

The extracellular acidification rate (ECAR) was measured as previously described using a Seahorse XF96 extracellular flux analyser (Seahorse Bioscience, North Billerica, MA). The two MEF cell lines were measured simultaneously with quadruplicate wells per cell line. Then, the entire experiment was repeated. To ensure equal cell numbers across the two cell lines, cells were seeded in XF96 cell culture plates coated with Cell-Tak (BD Biosciences, San Jose, CA) at 8 × 10^3^ cells/well and incubated at 37 °C for 1 hr in prepared medium (99% basic cell culture medium and 1% glutamine) before analysis. Insulin was added to the final concentration of 5 μg/ml. Bioenergetic profiling was performed by monitoring followed by sequential injection of the following inhibitors with the final concentrations indicated in parentheses: glucose (10 mM), oligomycin (1 μM), and 2-deoxyglucose (50 mM)^61^.

### Statistical analysis

The repeatability of the findings was confirmed by performing all experiments a minimum of three times. Continuous values are presented as the means and standard deviations (SDs). Statistical analyses were performed by using Microsoft Excel version 2007. IBM SPSS statistical software version 21 was used to perform a one-way ANOVA to analyse differences between groups. *p* < 0.05 (*) and *p* < 0.01 (**) indicate statistically significant Fand highly statistically significant differences, respectively.

## Additional Information

**How to cite this article**: Xu, Y. S. *et al*. STAT3 Undergoes Acetylation-dependent Mitochondrial Translocation to Regulate Pyruvate Metabolism. *Sci. Rep.* **6**, 39517; doi: 10.1038/srep39517 (2016).

**Publisher's note:** Springer Nature remains neutral with regard to jurisdictional claims in published maps and institutional affiliations.

## Supplementary Material

Supplementary Information
