# Supplementary material for: STAT3 Undergoes Acetylation-dependent Mitochondrial Translocation to Regulate Pyruvate Metabolism
Source: Sci Rep. 2016 Dec 22;6:39517. doi: 10.1038/srep39517 (PMC5177931; doi:10.1038/srep39517)

# STAT3 Undergoes Acetylation-dependent Mitochondrial Translocation to Regulate Pyruvate Metabolism

Yan S. Xu[1], Jinyuan J. Liang[1], Yumei Wang[2], Xiang-zhong J. Zhao1,[2], Li Xu[3], Ye-yang Xu[1], Quanli C. Zou[2], Junxun M. Zhang[2], Cheng-e Tu[2], Yan-ge Cui[2], Wei-hong Sun[2], Chao Huang[1,4], Jing-hua Yang[1,*,] Y. Eugene Chin[2,*]

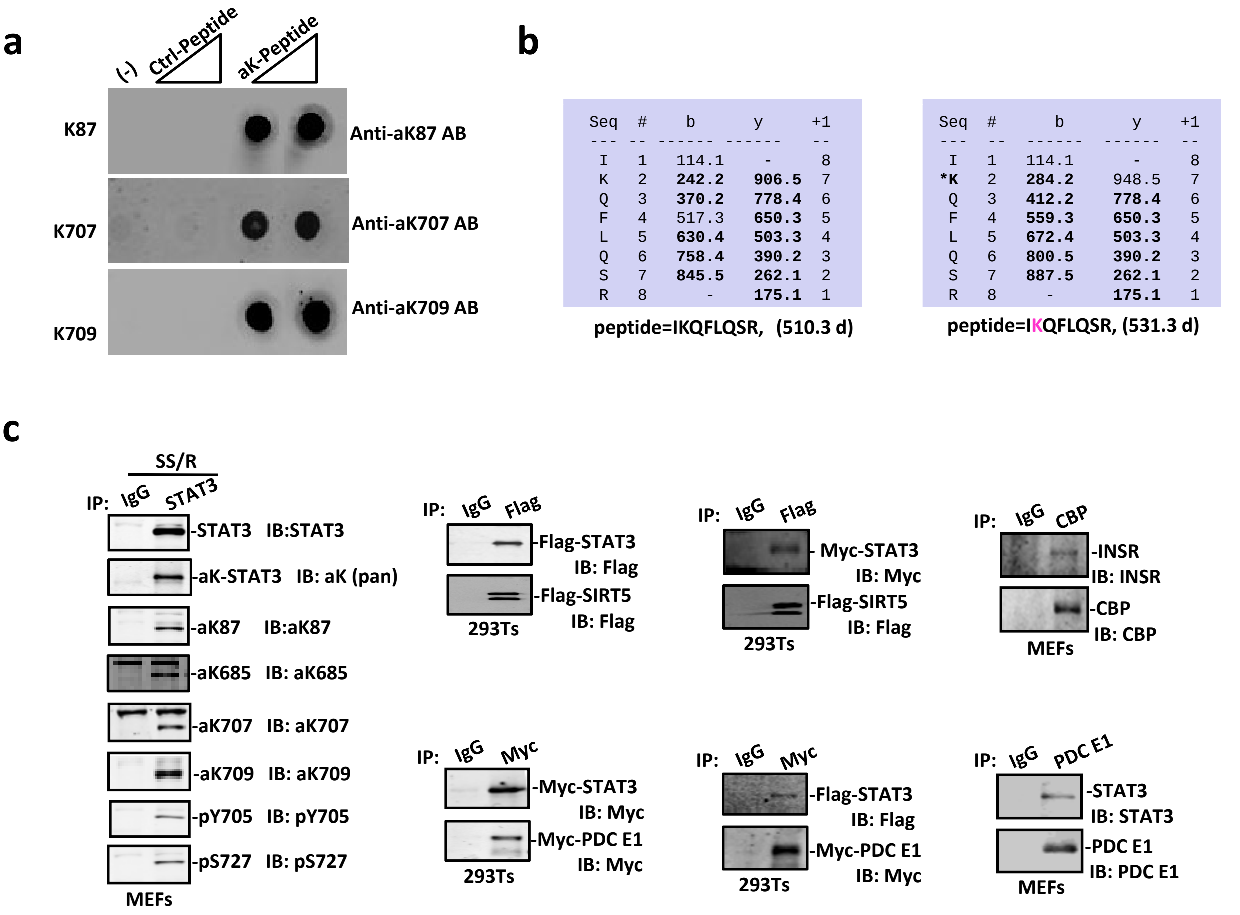


**Figure S1.** (a) Peptide slot blot analysis of the antibodies prepared for STAT3 acetylation on K87, K707 and K709. (b) y and b numbers of the mass spectrum in Fig. 2b. (c) IgG controls were detedted for Immunoprecipitation and co-immunoprecipitation experiments involved in this text.

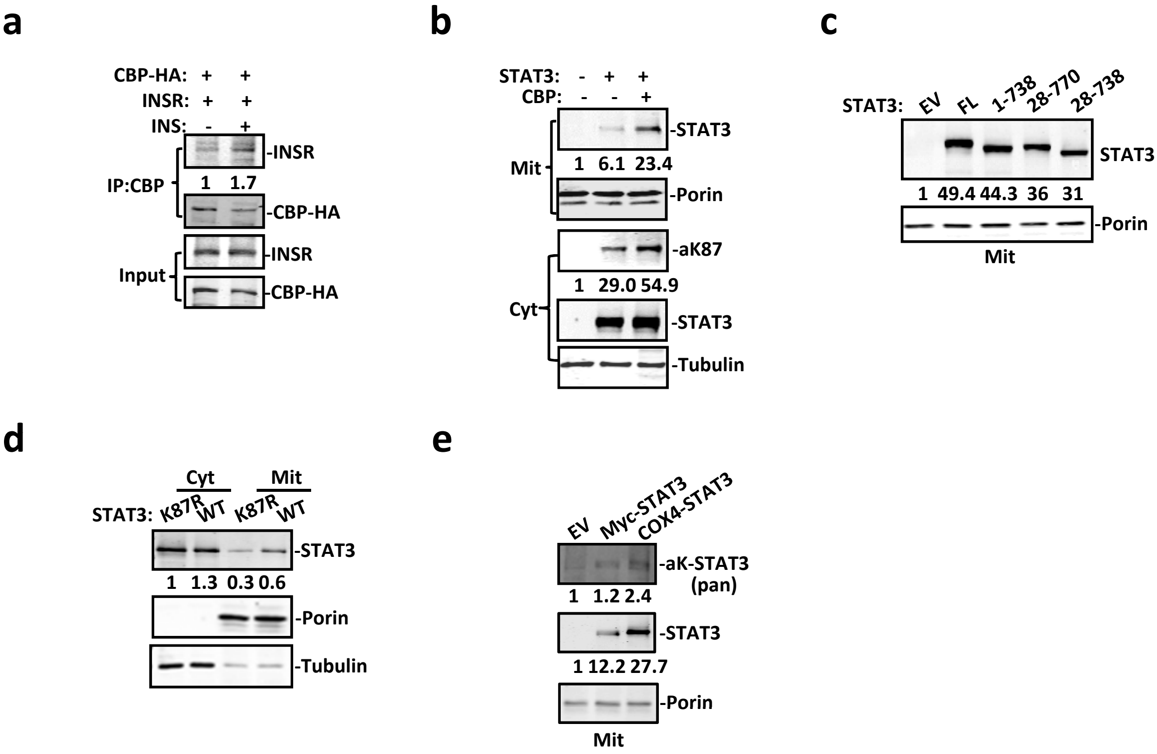


**Figure S2.** (a) INSR and CBP were overexpressed in 293T cells. HA-CBP was immunoprecipitated followed by Western blotting analysis with anti-INSR and anti-HA. The bottom panel gave the input of INSR and HA-CBP. (b) 293T cells were transfected with EV, STAT3, or STAT3 and CBP. STAT3 acetylation on lysine 87 was detected with a specific antibody via Western blotting. (c) 293T cells were transfected with EV, wild-type STAT3 and STAT3 with the N-terminus, C-terminus, or both N- and C-termini deleted (i.e., 1-738, 28-770, and 28-738). Mitochondria were isolated from the 293T transfectants and STAT3 and Porin levels were shown by Western blotting. (d) STAT3-K87R mutant or STAT3-WT was transiently transfected into 293T cells. Cytoplasmic and mitochondrial fractions were prepared for STAT3 localization analysis via Western blotting with anti-Myc. (e) Mitochondrial lysates were prepared for STAT3 acetylation analysis from 293T cells transfected with EV, STAT3 or Cox4-STAT3.

**Figure S3.** Full scans of western blotting data in all figures.

Fig. 1a

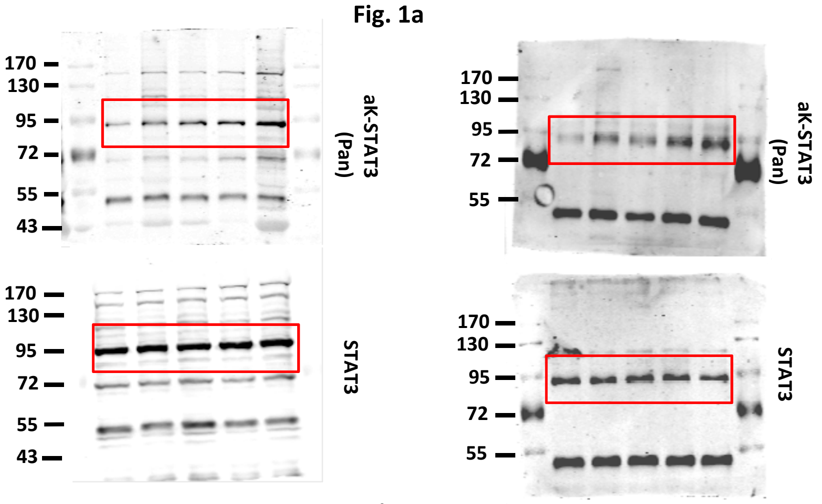


Fig. 1c

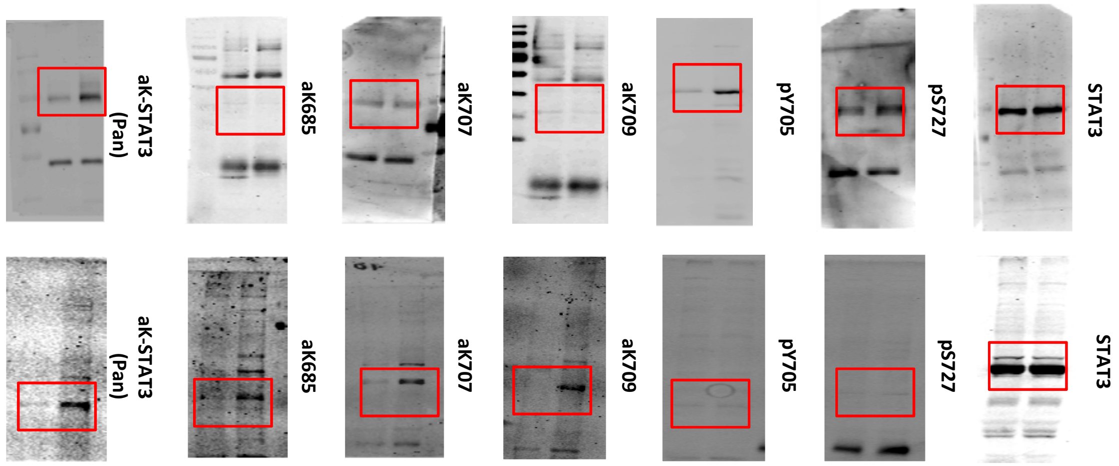


Fig. 1d

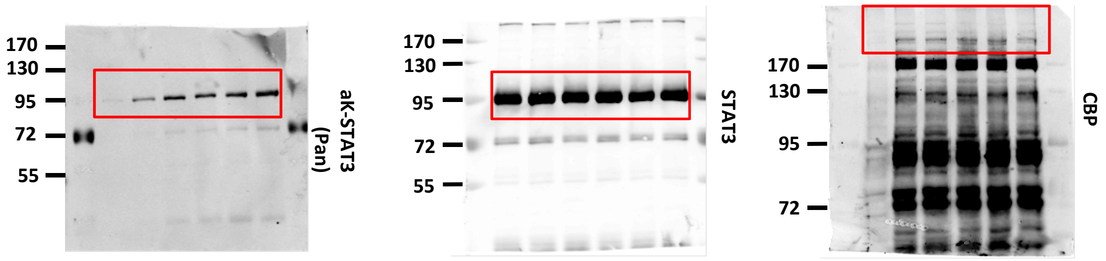


Fig. 1e

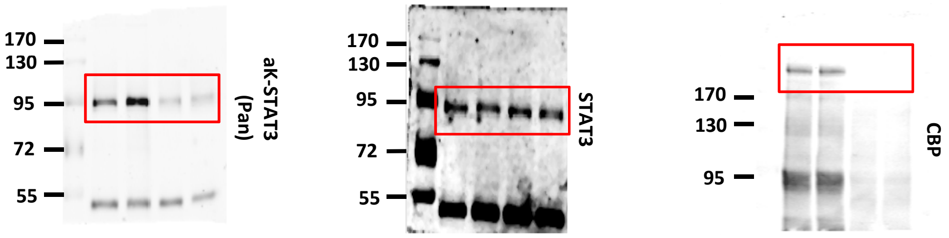

**Fig. 1f**

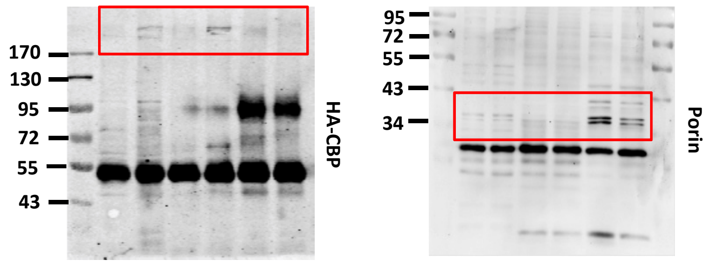


**Fig. 1g**

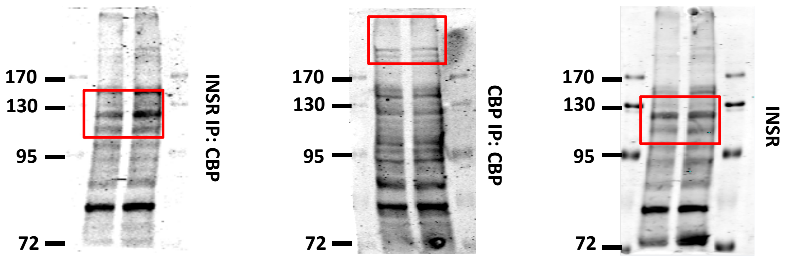


**Fig. 1h**

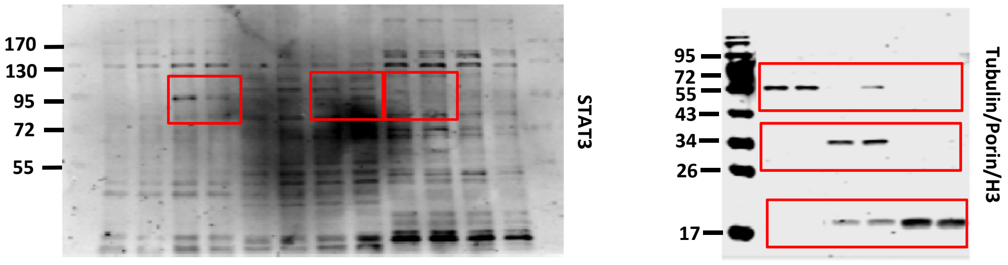


**Fig. 2a**

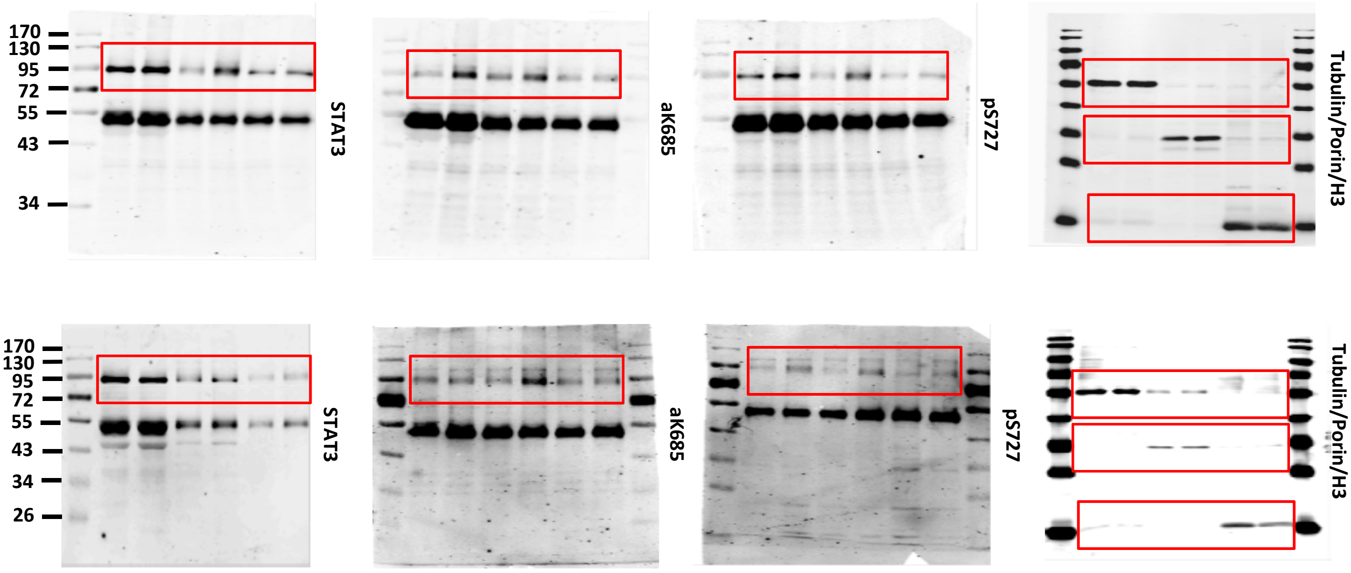

**Fig. 2b**

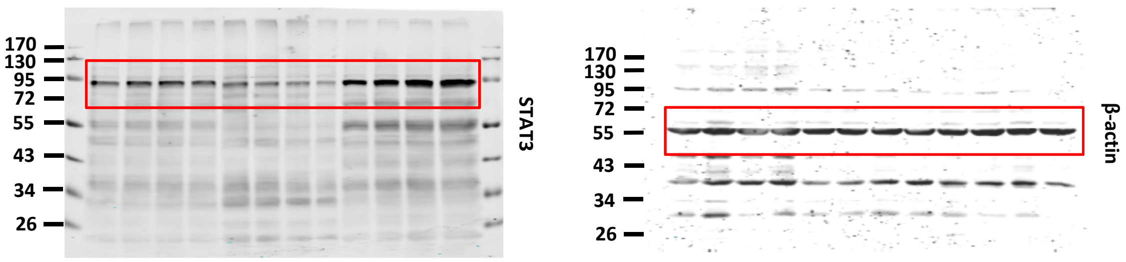


**Fig. 2d**

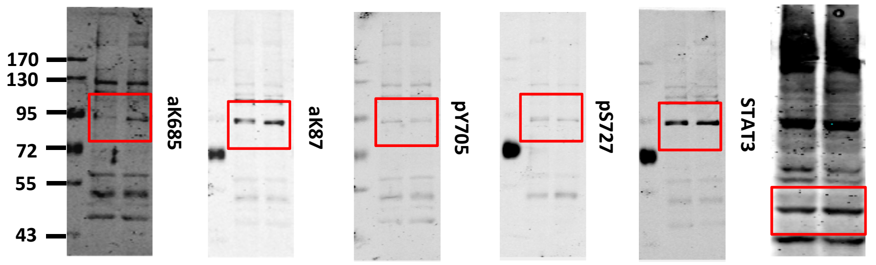


**Fig. 2e**

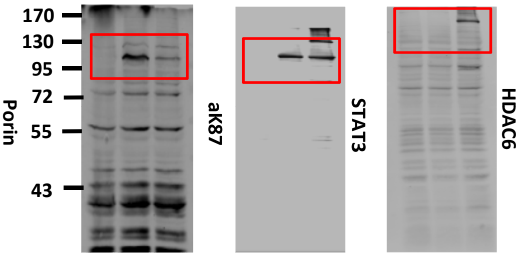


**Fig. 2f**

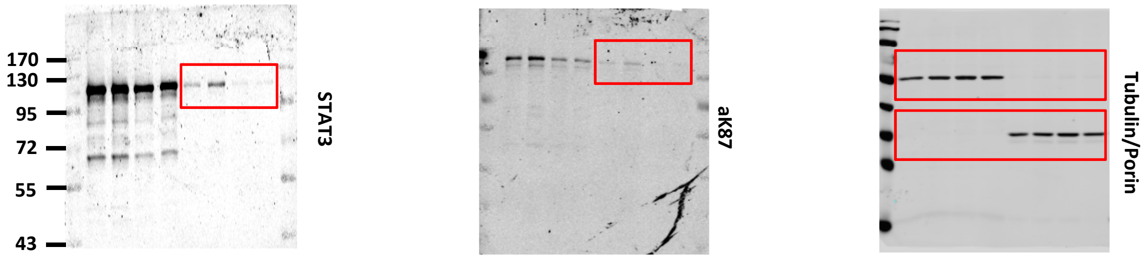


**Fig. 2g**

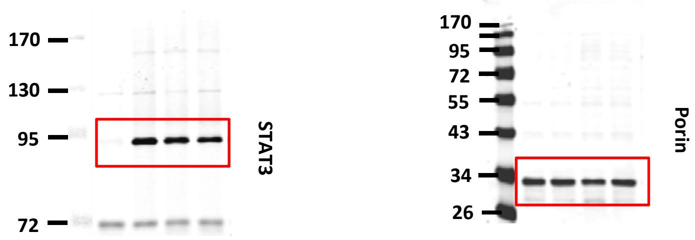


**Fig. 2h**

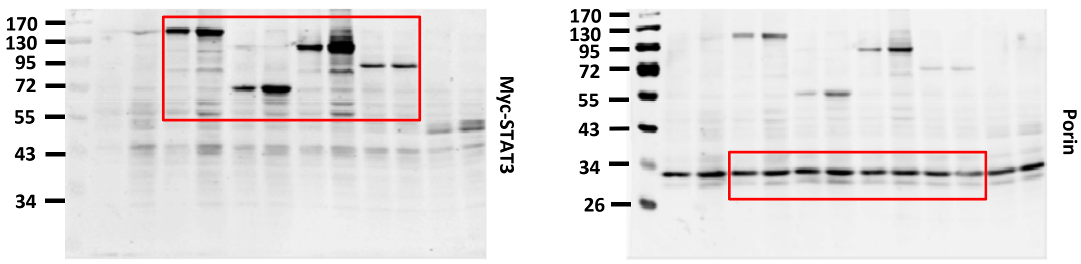

Fig. 2i

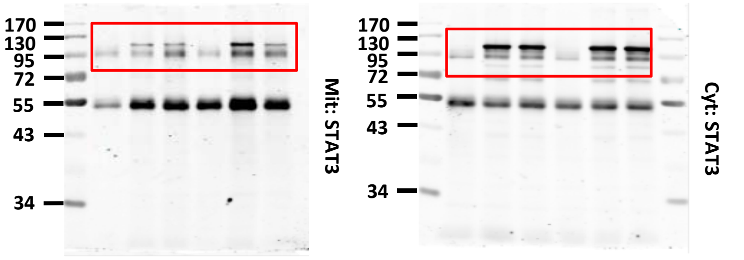


Fig. 2j

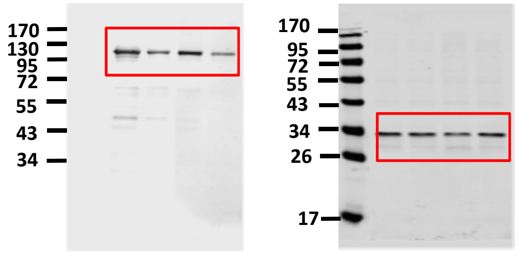


Fig. 3a

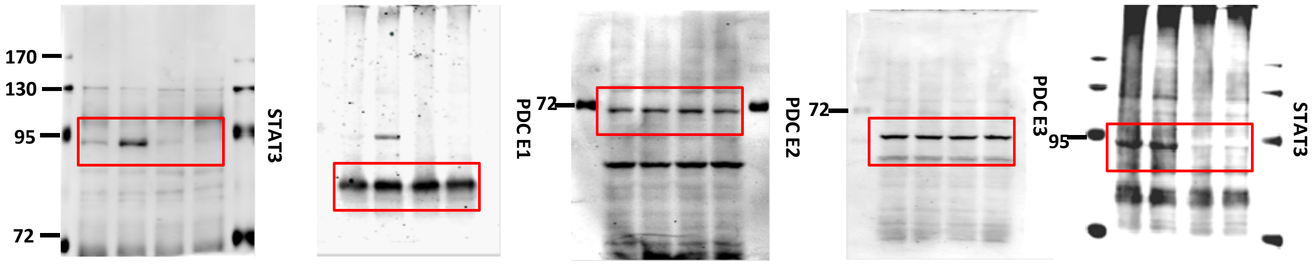


Fig. 3b

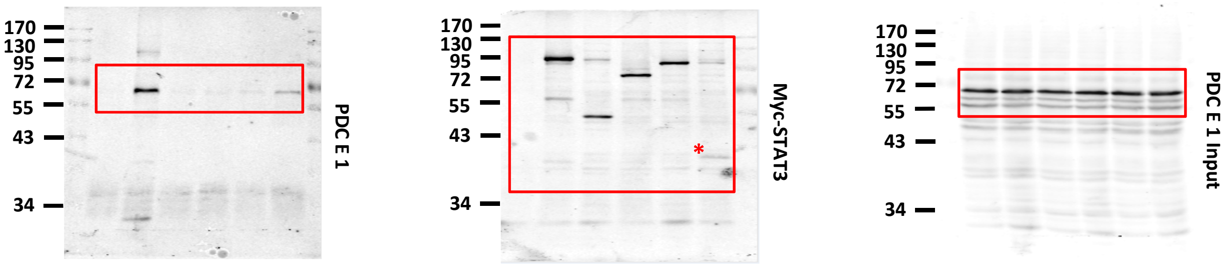


Fig. 3d

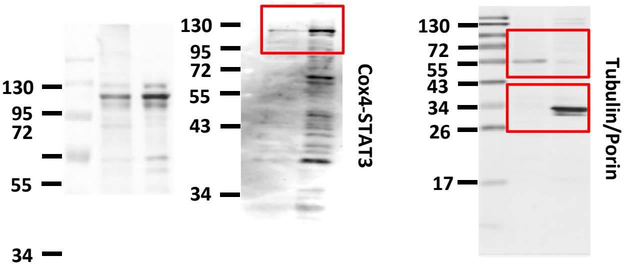


Fig. 3g

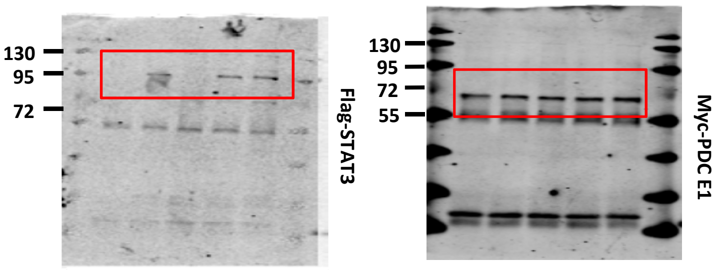


Fig. 4a

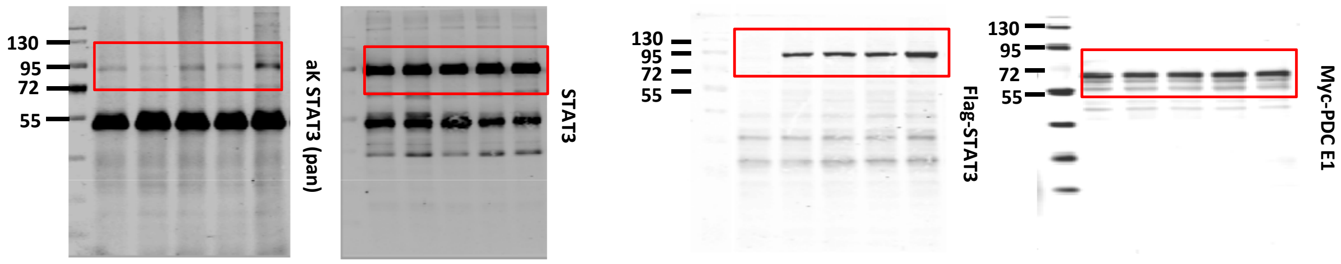

**Fig. 4b**

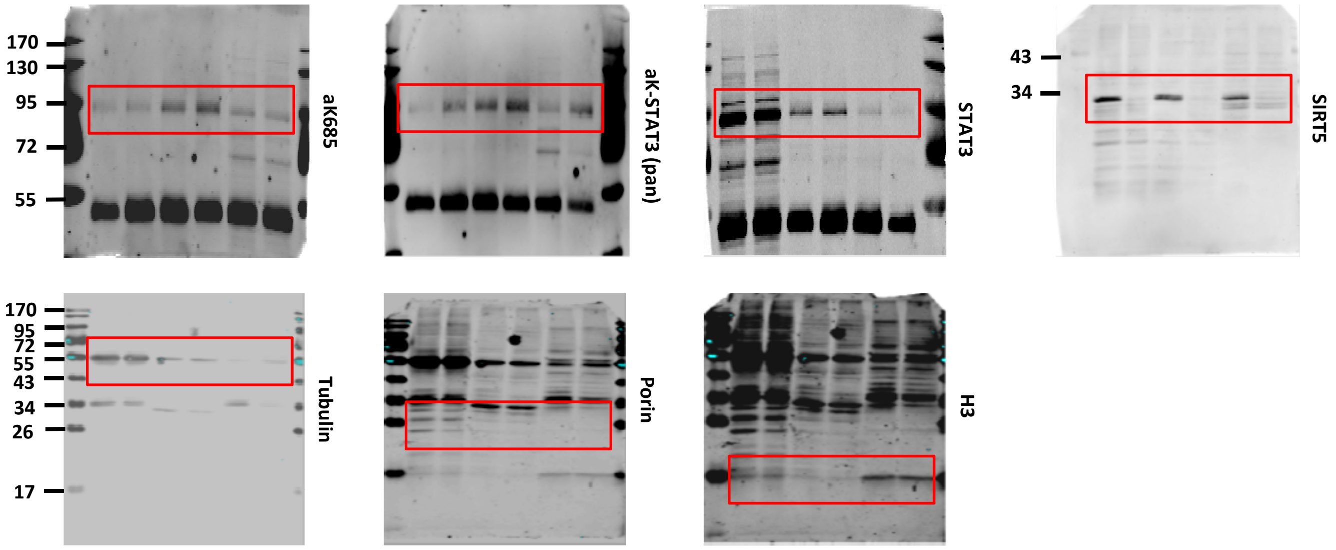


**Fig. 4c**

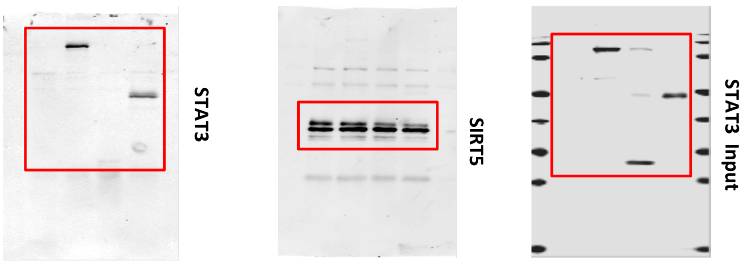


**Fig. 4d**

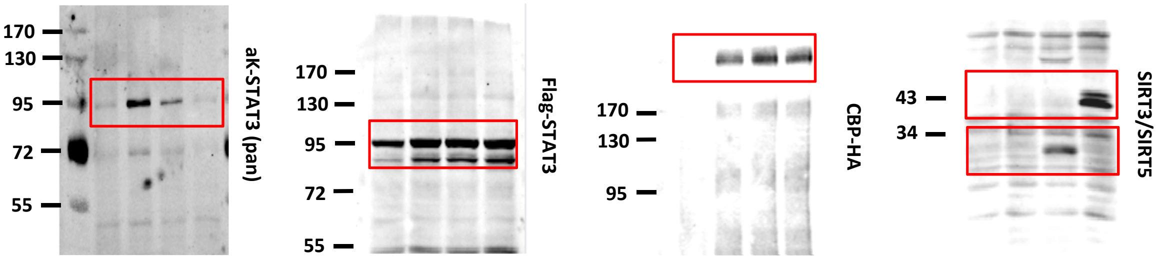


**Fig. 4e**

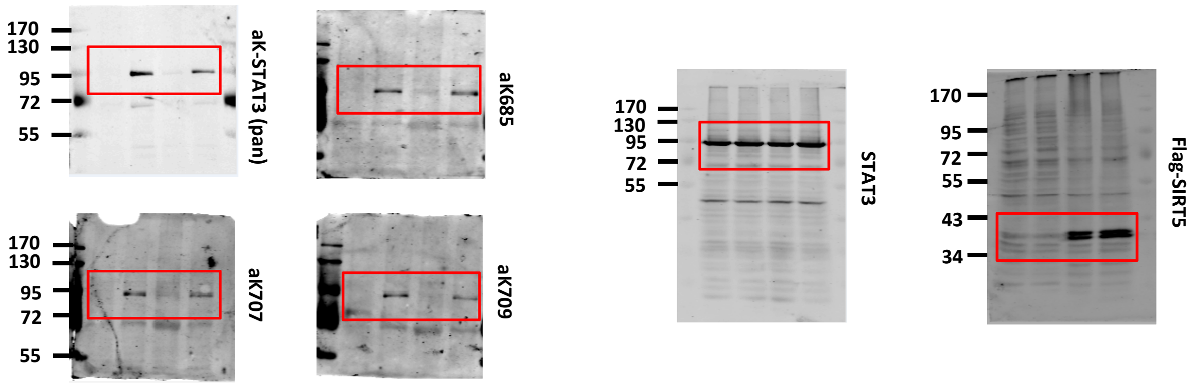

## Fig. 4g

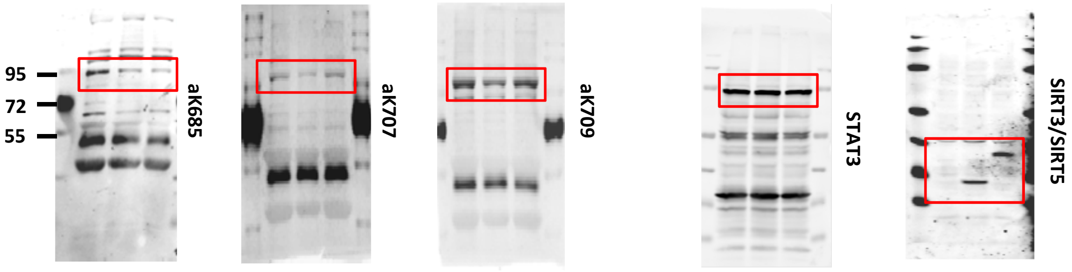


## Fig. 7b

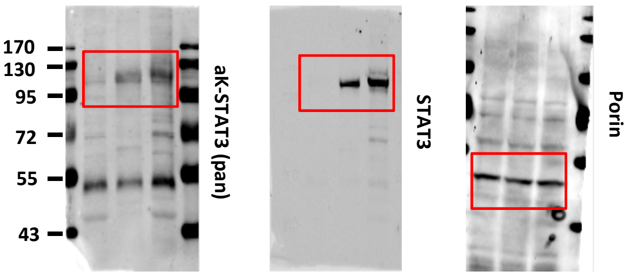


## Fig. 7f

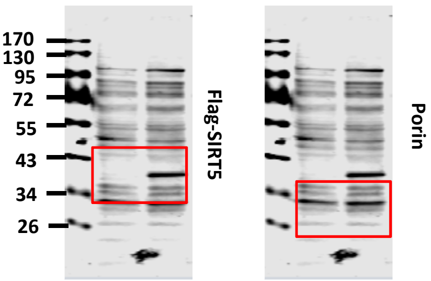


## Fig. 7e

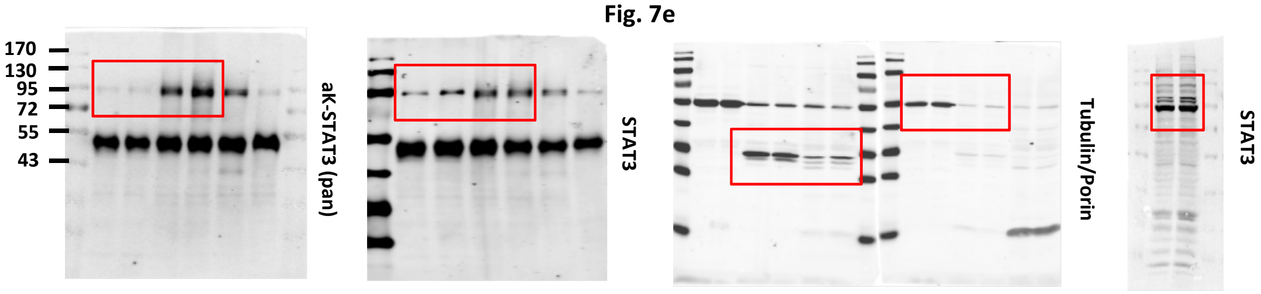


## Fig. 7j

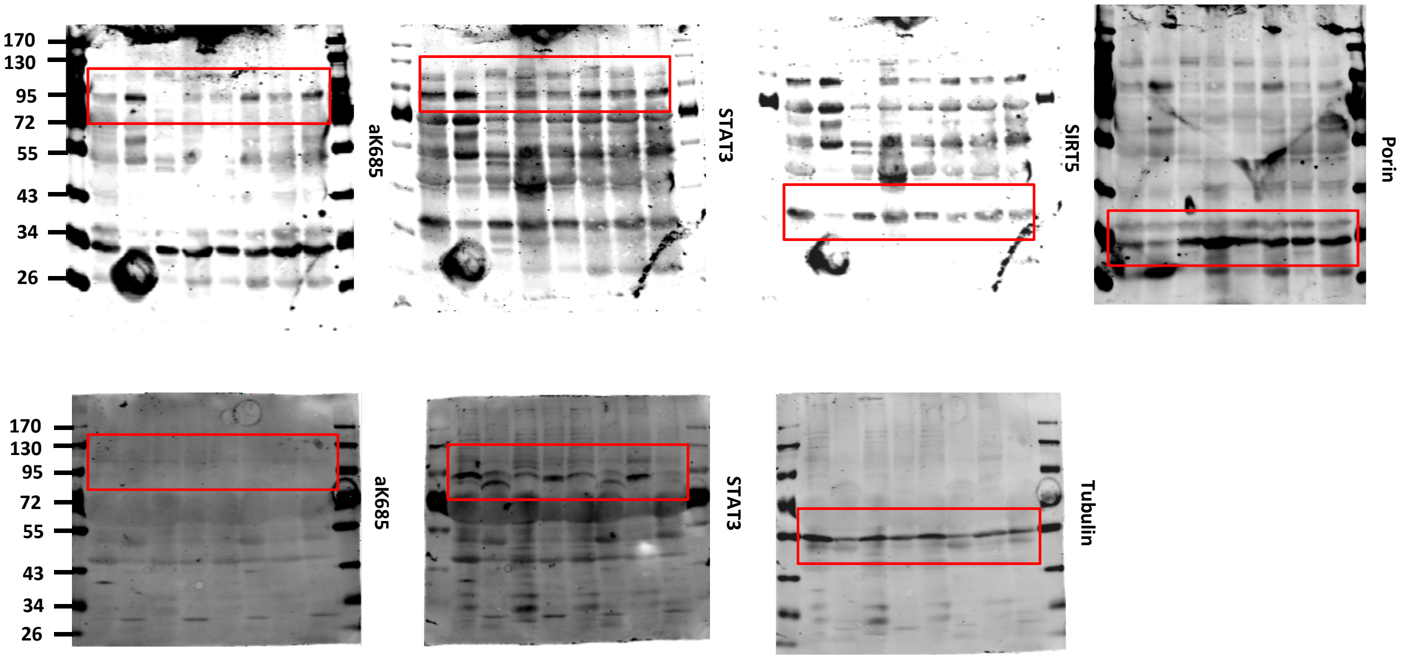

Supplementary Fig. 1c

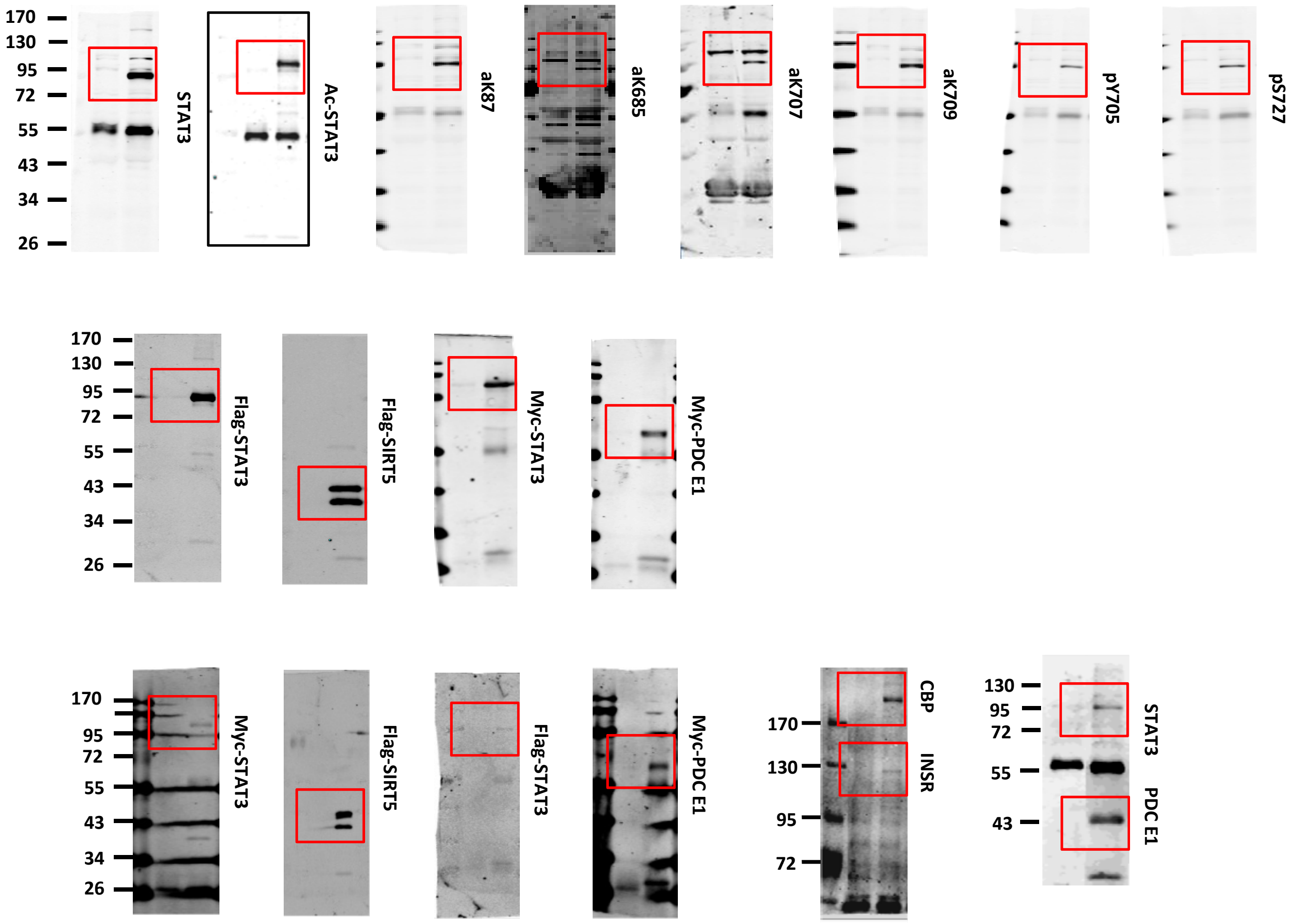

## Supplementary Fig. 2a

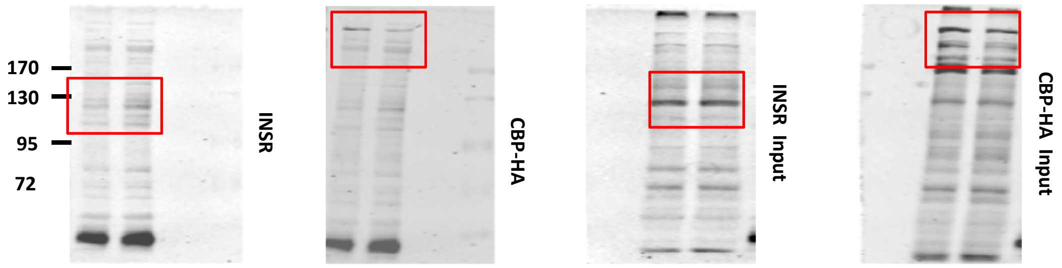


## Supplementary Fig. 2b

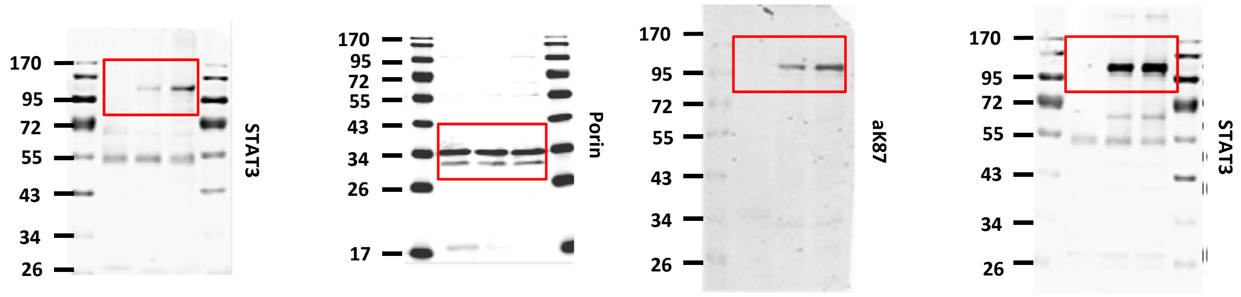


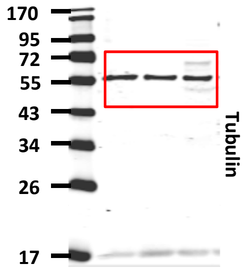


## Supplementary Fig. 2d

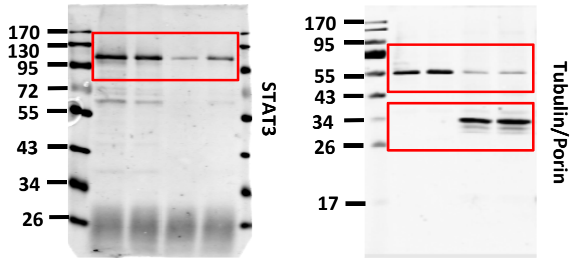


## Supplementary Fig. 2c

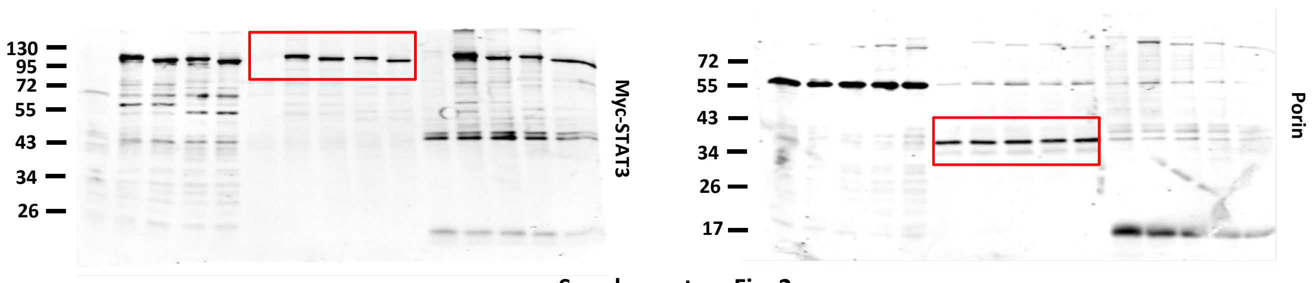


## Supplementary Fig. 2e

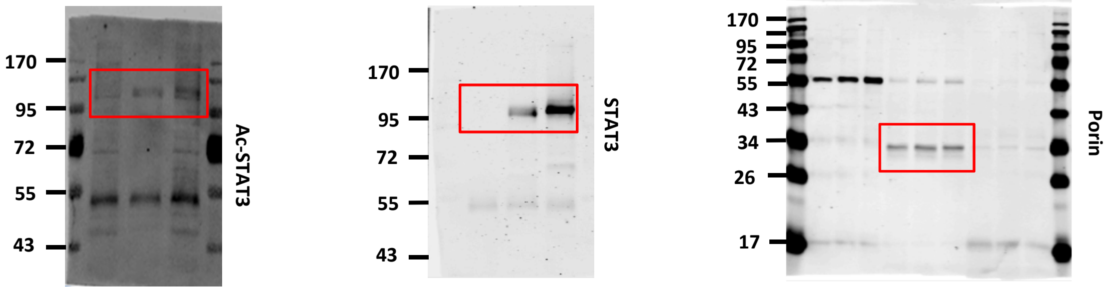

Supplement: Supplementary Information [file srep39517-s1.pdf]
